# Aggregation induced emission behavior in oleylamine acetone system and its application to get improved photocurrent from In_2_S_3_ quantum dots

**DOI:** 10.1038/s41598-020-76703-0

**Published:** 2020-11-12

**Authors:** Subramaniam Ramya, Devaraj Nataraj, Sangameswaran Krishnan, Sellan Premkumar, Thankappan Thrupthika, Arumugam Sangeetha, Kittusamy Senthilkumar, T. Daniel Thangadurai

**Affiliations:** 1 Quantum Materials and Devices Laboratory, Department of Physics, Bharathiar University, Coimbatore, Tamil Nadu 641046 India; 2 UGC-CPEPA Centre for Advanced Studies in Physics for the Development of Solar Energy Materials and Devices, Department of Physics, Bharathiar University, Coimbatore, Tamil Nadu 641046 India; 3 Molecular Quantum Mechanics Laboratory, Department of Physics, Bharathiar University, Coimbatore, Tamil Nadu 641046 India; 4 School of Chemistry and Chemical Engineering, Tiangong University, Tianjin, 300387 China; 5 School of Material Science and Engineering, Tiangong University, Tianjin, 300387 China; 6 Department of Nanoscience and Technology, Sri Ramakrishna Engineering College, Coimbatore, Tamil Nadu 641022 India

**Keywords:** Surface chemistry, Energy science and technology, Materials science, Nanoscience and technology, Optics and photonics

## Abstract

Blue emission giving nanoscale molecular clusters of Oleylamine–Acetone system was formed by an aging assisted hydrogen bond formation between the interacting molecular systems, at room temperature. The as-formed nanoscale molecular clusters were found to be self-assembled into flower-like aggregates and shifted the emission wavelength to red colour depicting an exciton delocalization in the aggregate system. Interestingly aging process has also produced imine type binding between Oleylamine and Acetone due to the condensation reaction. The experimental conditions and formation mechanism of hydrogen bond assisted Oleylamine–Acetone molecular aggregates and imine bond assisted Oleylamine–Acetone is elaborated in this paper in a systematic experimental approach with suitable theory. Finally we have introduced this Acetone assisted aging process in In_2_S_3_ QD system prepared with Oleylamine as functional molecules. It was found that the aging process has detached Oleylamine from QD surface and as a consequence In_2_S_3_ QD embedded Oleylamine–Acetone aggregates was obtained. When this In_2_S_3_ QD embedded molecular cluster system was used as an active layer in a photo conductor device then a maximum photo current value of the order of milli Ampere was obtained. The surfactant molecules normally inhibit the charge transport between QD systems and as a result it is always problematic to have the functional molecules in the QD based transport devices. Our approach has a solution to this problem and the present paper discusses the outcome of the results in detail.

## Introduction

The study on “Molecular aggregates”^1–4^ is an exciting topic due to its interesting coulombic as well as vibrational coupling between nearby molecules leading to the better exciton transport within the aggregate^5,6^. Michael Kasha, American photochemist has clearly explained the concept of photophysics in molecular aggregates. According to kasha’s rule, molecular aggregates can be classified into two types such as H-aggregates and J-aggregates^5,7,8^. H-aggregate is the aggregate in which transition dipoles of neighbouring molecules are aligned in a parallel fashion, exhibiting hypsochromic shift in the absorption spectrum compared to monomer absorption^9,10^. On the other hand, when transition dipoles of neighbouring molecules are aligned in a head to tail manner, it exhibits bathochromic shift in absorption spectrum compared to monomer absorption which is coined as J-aggregates^9,11,12^. Packing of molecules to form aggregate is highly related to intermolecular interactions between the molecules. The intermolecular interactions in the form of magnetic dipole moment, electric dipole moment, pi–pi stacking, halogen^13^ or hydrogen bonding allows ordered structure of molecules^14^. Among which hydrogen bonding triggered self-assembly is very interesting one^15–20^. High boiling point of water arises from OH…HO type hydrogen bond in the system^21^. NH…O=C type hydrogen bond exist in DNA system make them semiconducting^22,23^. Hydrogen bonded pigment indigo is a very good organic semiconductor. NH…O=C type hydrogen bond with neighbouring molecules along one particular crystal axis and π-stacking along perpendicular direction leads to higher mobility in indigo. Optical absorption spectra of most of the hydrogen bonded pigments undergo extensive bathochromic shift from dilute solution to the solid state. Hydrogen bond enhanced delocalization effect^24–26^ was briefly explained in ϒ-quinacridone^27^. This pigment is used as an active layer in photovoltaic device and shows considerable external quantum efficiency of nearly 10%. Herewith we have illustrated the NH…O=C type hydrogen bond interaction found between Oleylamine and Acetone and studied their self-assembling nature upon aging for the first time.

Oleylamine^28–30^ is a well-known capping ligand^31^ used for the synthesis of various metal and semiconducting nanomaterials^30,32,33^. This molecule has received much research attention in the area of phosphine-free growth of colloidal quantum dots^34,35^. Oleylamine can be used as a passivating layer in silicon solar cell to enhance the device efficiency^36^. Doping of Self-assembled Oleylamine networks^37^ in MoS_2_ transistor can increases the carrier concentration from 0.7 × 10^12^ cm^−2^ to 1.9 × 10^13^ cm^−2^. Oleylamine belongs to the family of primary aliphatic amines and the chemical structure of Oleylamine is similar to that of Oeic acid except the end group COOH (the end group is NH_2_ in Oleylamine). It is well known that amine–carbonyl type hydrogen bonding is much studied in pigments like Indigo, tyrian purple, quinacridone, epindolidione and biomolecules such as DNA and proteins^16^. Because Oleylamine contains two hydrogen atoms covalently bonded to the nitrogen atom, it can easily form hydrogen bond with neighbouring molecules. With this basic knowledge, here we have chosen carbonyl group containing molecule Acetone and amine group containing molecule Oleylamine and studied their possibility to form amine–carbonyl type intermolecular hydrogen bonding interaction. Interestingly, aging assisted formation of hydrogen bond in the Oleylamine–Acetone was found at room temperature and that leads to the formation of nanoscale molecular clusters and their flower like self-assemblies. The present paper gives an insight into the formation of such a structures and their application in a systematic approach. Interestingly we have also observed the room temperature formation of imine type interaction between Oleylamine and Acetone molecules during the aging process and that was due to the condensation reaction between Oleylamine and Acetone across the interface between solute and solvent. This process has converted Oleylamine into Oleylimine. By optimizing the ratio between Oleylamine and Acetone an interface free condition was set to avoid the formation of Oleylimine and as a result hydrogen bond assisted molecular cluster/aggregates were only formed.

Nanoscale cluster to macro scale aggregates and their reversible process was controlled by changing the dilution level of the cluster system and as a result rapid reversible photoluminescence behavior was also noticed. This paper discusses about the reversible emission behavior of the molecular system and the formation of mechanism of the molecular clusters in detail. Finally we have introduced this strategy, that is, Acetone assisted aging process in In_2_S_3_ quantum dot system prepared with Oleylamine as surfactant molecule, and this is in order to understand the consequences of aging process in photo current generation in In_2_S_3_ QD system. Interestingly the aging process has removed the Oleylamine surfactant molecules from In_2_S_3_ QDs. As evidence slightly size enlarged In_2_S_3_ QDs were found to be coexisting with Oleylamine–Acetone molecular cluster system. It was found that the molecular cluster system worked as conducting matrix, and thus helped to improve the overall photo current generation to few milli amps. In the present paper the formation conditions of hydrogen bond assisted Oleylamine–Acetone molecular clusters/aggregates and covalent interactions between the molecules will be discussed first and followed by In_2_S_3_ QD preparation/device results will be discussed.

## Experimental methods

### Chemicals

Oleylamine (≥ 98%, Primary amine) purchased from Aldrich and Acetone (99%) obtained from Loba chemicals were used in this study.

### Immisible Oleylamine–Acetone hybrid system with low Acetone concentration

A 5 ml of Oleylamine mixed with 2 ml of Acetone stored in centrifuge tubes initially looked like transparent in nature and after ten minutes it was converted into whitish cloud solution. Then the solution was separated into two layers when the aging process proceeds for four weeks. The upper part seems to be yellowish in colour and the lower part seems to be colourless. When we excite the sample with UV laser, then we have attained dual colour emission from the sample in such a manner that the upper part looks yellow in colour and the lower part looks blue in colour.

### Miscible Oleylamine–Acetone hybrid system with high Acetone concentration

A 5 ml of Oleylamine mixed with 10 ml of Acetone exhibited transparent colour during the day of mixing, then every week its colour changed from pale yellow to dark red after one month. Almost similar experiments were conducted with different ratio as mentioned in the main text.

### In_2_S_3_ quantum dot preparation

Indium Chloride (99.9%, Sigma Aldrich), Elemental Sulfur (99.9%, Sigma Aldrich) were used as a source material for In^3+^ and S^2−^ ions respectively. Dodecanthiol (99%, Sigma Aldrich) and Oleylamine (98%, Aldrich) were used for the capping purpose. Acetone (99%, Loba Chemicals) was used for aging study. A 0.001 mol of indium chloride was dissolved in 1-Dodecanthiol and stirred under Argon atmosphere at 110 °C for half an hour. Elemental Sulfur was dissolved in Oleylamine and stirred under vacuum at 90 °C for half an hour. After that elemental sulfur solution was quickly injected into indium chloride solution and mixed under Argon atmosphere for 20 min to grow indium sulfide quantum dots. As obtained quantum dots were purified by means of using equimolar ratio of Hexane (Solvent) and Methanol (Antisolvent) under centrifugation at 5000 rpm for 30 min. As prepared Oleylamine and 1-Dodecanthiol capped blue emitting In_2_S_3_ quantum dots were dissolved in Acetone and kept under aging for 1 month.

## Results and discussion

**Figure 1:**
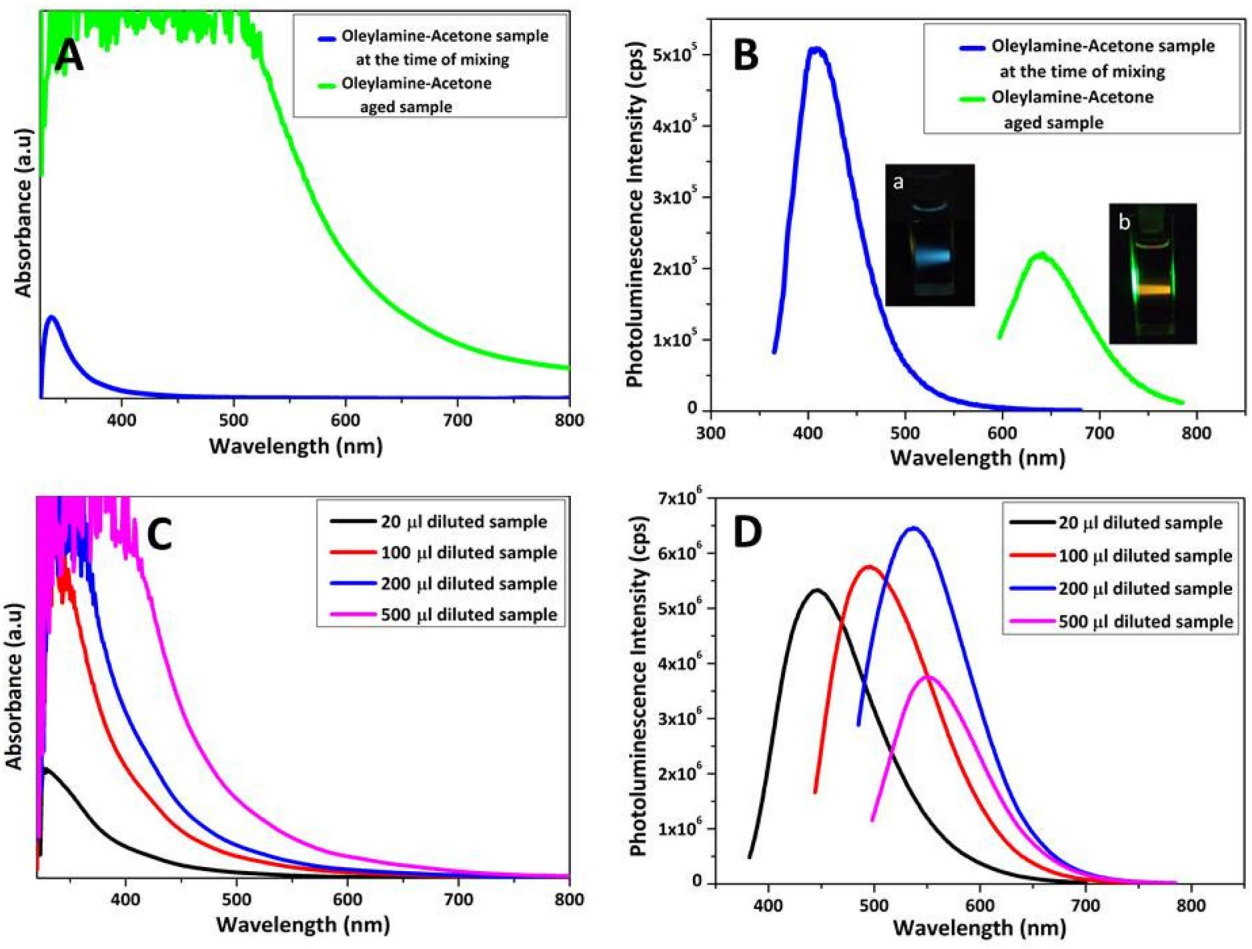
Optical properties of Oleylamine–Acetone sample. (**A**) Oleylamine–Acetone sample at the time of mixing shows absorption maximum at 350 nm in the UV region, but after an aging process for 1 month it shows a red shifted absorption maximum at 585 nm. (**B**) The corresponding samples emission spectra show red shifted wavelengths. The images (a, b) in the insect of (**B**) denotes the blue and orange emission colours from Oleylamine–Acetone sample, respectively corresponding to the time of mixing and aged samples, under UV and Green wavelength excitations. Figures (**C**,**D**) are the absorption and emission spectra recorded from Oleylamine–Acetone aged sample at different levels of dilution. The spectra have shown the dilution caused shift in both the absorption and emission peak positions. As dilution increases then a blue shifted absorption and emission wavelength were noticed.

**Figure 2:**
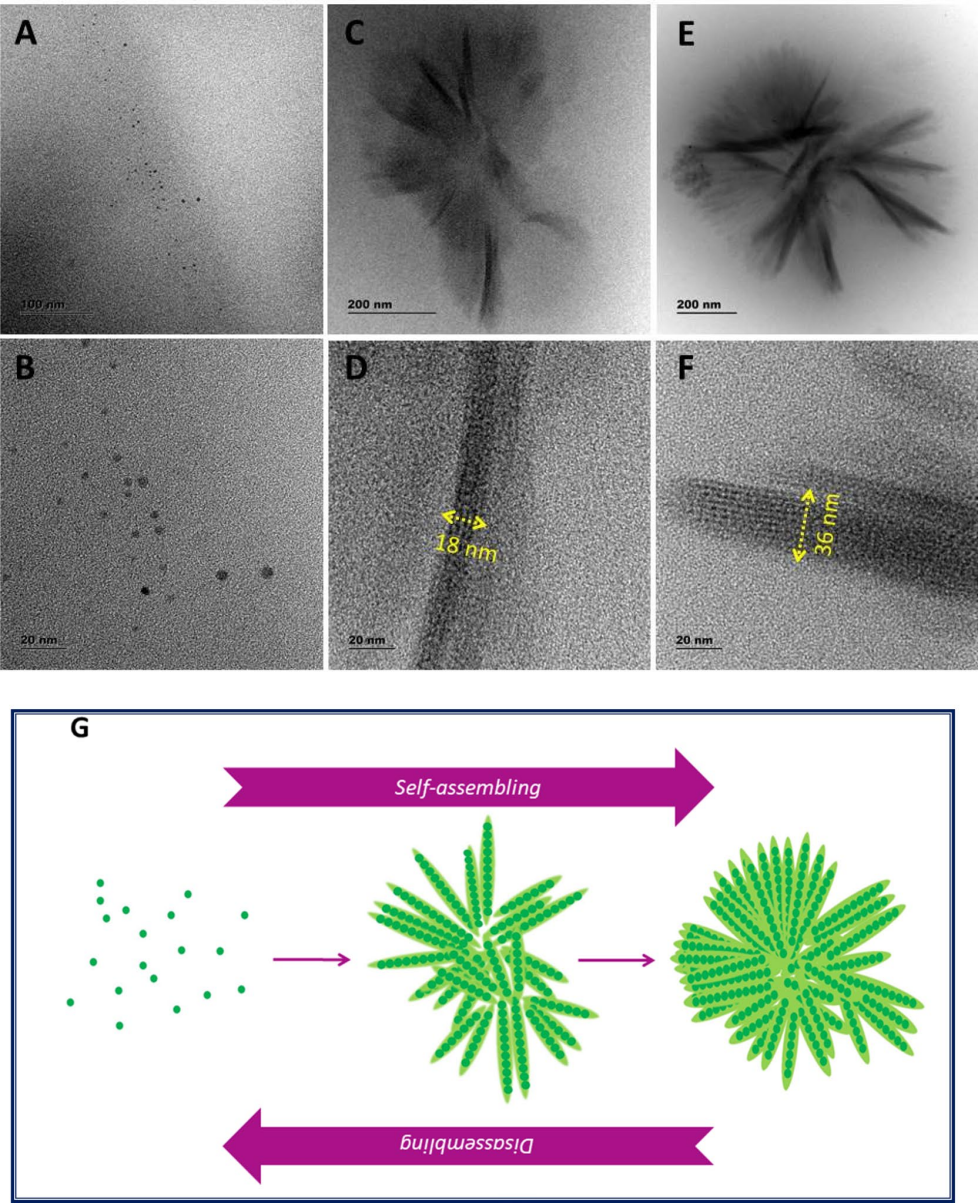
HRTEM images of Oleylamine–Acetone aged sample recorded at different dilutions. The solvent Acetone volume was kept to be constant as 5 ml and to which different volumes of aged sample (20 μl, 100 μl, 200 μl and 500 μl) was added to produce different levels of dilutions. (**A**) is the image from 20 μl diluted sample, (**C**) 100 μl diluted sample, (**E**) 200 μl diluted sample at the scale bar of 200 nm. (**B**,**D**,**F**) are higher magnification images at the scale bar of 20 nm. Aging assisted formation of nanoscale molecular (Oleylamine–Acetone) cluster can be visualized in the 20 μl diluted sample. (**G**) Schematic illustration denoting the self-assembly and Disassembly of flower-like aggregates in the concentrated and diluted states respectively.

A 5 ml of technical grade Oleylamine (C_18_H_35_NH_2_) obtained from Sigma Aldrich was mixed with 10 ml Acetone (CH_3_COCH_3_) and the resulting sample’s optical absorption and emission spectra were recorded before aging. The absorption and emission^38^ maximum were at 350 nm and 410 nm respectively for this sample as shown in Fig. 1A,B. The as prepared mixture solution was then kept under aging for about 30 days and found to be converted into a thick red colour solution. The corresponding samples absorption and emission maximum were found to be strongly red shifted to 585 nm and 640 nm respectively. Further the emission intensity of this aged samples was found to be quenched. The dark red solution was diluted by adding Acetone to look for an enhanced red emission. But surprisingly due to dilution effect we have obtained blue shifted emission spectra. An effort was taken to study this reversible blue shift by taking different micro liters of aged solution and diluting them in a fixed volume of Acetone (5 ml) and by doing so it was possible to shift the emission maximum towards blue side in a controlled manner as in (Fig. 1C,D). For example, when 500 μl red solution was diluted in 5 ml Acetone then an emission maximum at 555 nm corresponding to the excitation at 470 nm was observed. When 200 μl red solution was diluted then emission spectra has shown slight enhancement in emission intensity and blue shifted emission at 537 nm. For 100 μl red solution, the dilution has resulted blue shifted emission at 494 nm. Compared to 100 μl diluted sample, when 20 μl aged sample is diluted then it was possible shift the emission to 444 nm corresponding to the excitation at 367 nm. Thus, by controlling the dilution level preciously it was possible to fix the emission wavelength to a desired value and therefore a continuous tunability of emission wavelength was possible in this molecular solution. Such an observation is similar to the one from quantum dots, where emission tunability is done by varying the size^39^. By varying the size of the quantum dots, quantum confinement level was controlled to tune different emission wavelengths. In our case the samples are not QDs, but molecular solution and therefore the possibility of forming molecular clusters/aggregates could be the main reason behind the observed emission shift. In the case of molecular system, aggregation induced emission^40–42^ (AIE) and aggregation caused quenching (ACQ) are the two important phenomenon that occur in many of the organic molecules. Aggregation of fluorophore can yield any of the following emission characteristics in comparison to that of its diluted state (i) Quenching of fluorescence intensity. (ii) Enhancement of fluorescence intensity and unchanged fluorescence intensity. If the fluorescence intensity of aggregates is enhanced in comparison to that of dilution state then the process is called aggregation induced emission^43–46^. In contrast, if the fluorescence intensity of aggregates is reduced in comparison to that of dilution state then the process is called aggregation caused quenching. Some of the fluorophores exhibited aggregation induced redshifted emission (AIRSE)^43,47^. Herewith we have obtained the aggregation induced redshifted emission at first through aging process and a rapid dilution assisted reversible blue shifted emission. The observed photoluminescence behaviour clearly indicates a fact that there is a possibility of forming some types of clusters/aggregates and dis-aggregates. In order to find out the correlation between the change in the emission wavelength and micro structure of the samples, we have subjected the samples to HRTEM analysis (Fig. 2). The 20 μl diluted (in 5 ml Acetone) sample has exhibited spherical shaped particles having an average size of 3 nm. In the case of 100 μl diluted sample, we have obtained micron sized aggregates having a flower like morphology as shown in Fig. 2E. A close look into this micro structure has revealed a beautiful stacking arrangement of nanoparticles as shown in Fig. 2D. The stacking pattern has extended over an average lateral distance of 180 nm and width of 18 nm. The structural observation clearly tells the fact that the fundamental building block of the flower like pattern is nanosized particles and those nanoparticles were nothing but could be the molecular clusters made of Oleylamine–Acetone complex system. A similar flower like morphology with denser stacking of nanoparticles with an average lateral distance of 390 nm and width of 36 nm was obtained from 200 μl diluted sample (Fig. 2F).Thus from the morphological analysis of the samples we can understood the following facts. During aging process Oleylamine–Acetone interaction is established leading to the formation of nanoscale heterotype molecular clusters at first and then their selfassembly by Ostwald ripening, leading to the formation of flower-like aggregation. The formation of stable nanoscale sized molecular clusters took several days thus requiring an aging process, but the as formed clusters self-assembled into flower like aggregates immediately. When the well grown aggregates becomes responsible for the red emission, the nanoclusters were becoming responsible for blue emission. When the molecular structure was switching between these two extreme ends through a change in dilution process then the emission maximum was shuttling between these two wavelength regions. More importantly when nanocluster concentration of the diluted sample was increased then we have noticed immediate red shift, does not requiring any aging process. This observation clearly indicated a fact that the aging process is required to form nanoscale clusters, and once they are formed then the formation of flower shaped aggregates and dis-aggregates are faster process. It is therefore a rapid red to blue (or) blue to red emission wavelength tunability was possible by varying the dilution level of the solution.

**Figure 3:**
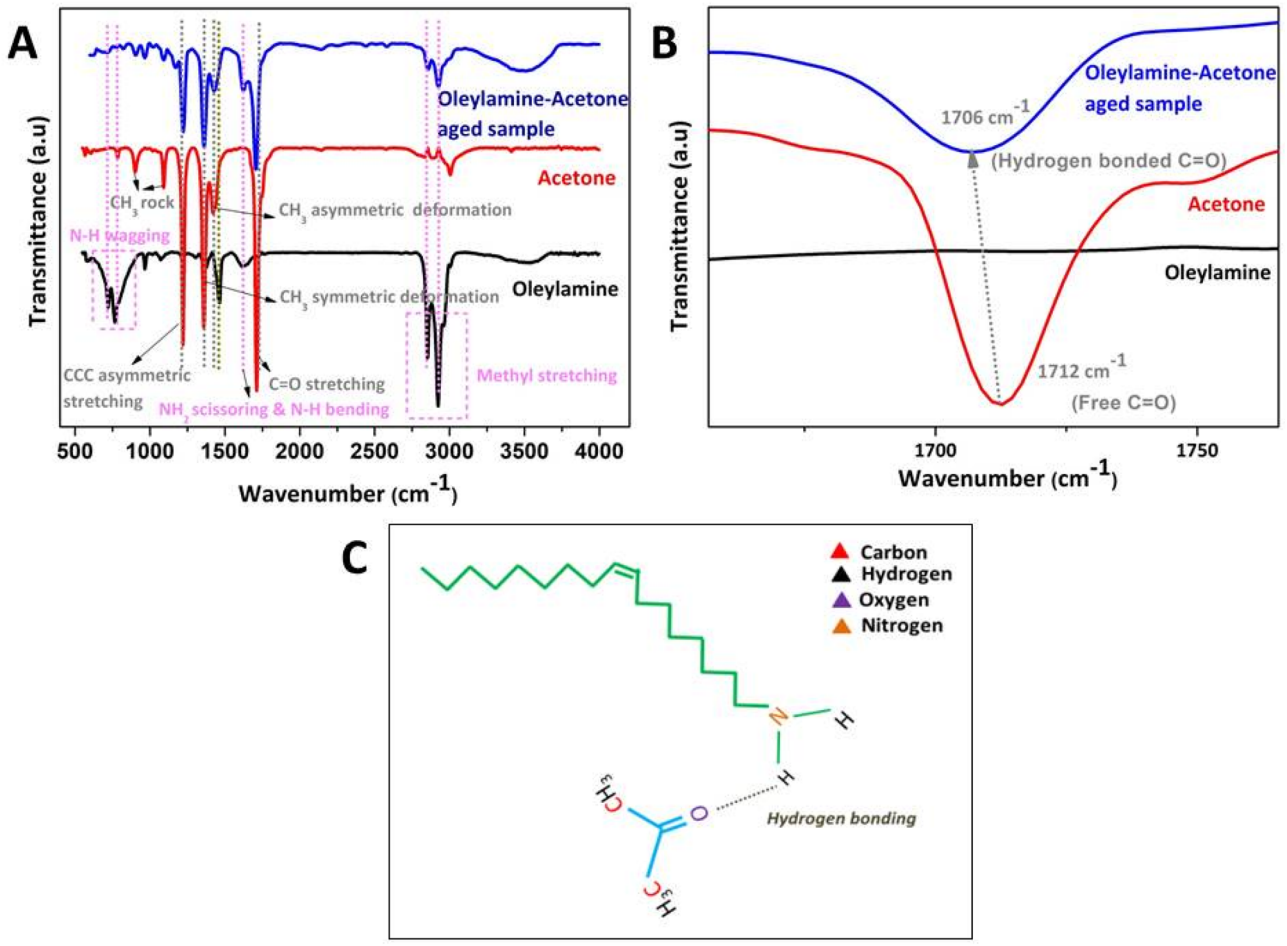
(**A**) FTIR spectrum for Oleylamine, Acetone and Oleylamine–Acetone aged sample. (**B**) An enlarged view of the FTIR spectra showing a red shift in C=O band position from the Oleylamine–Acetone aged sample, in comparison to that of Acetone and it confirms the hydrogen bond formation between Acetone carbonyl group and Oleylamine amine group. (**C**) Schematic diagram illustrating the formation of hydrogen bond interaction between Oleylamine and Acetone molecules.

In order to know about the vibrational level changes upon the formation of nanoscale molecular clusters and their self-assemblies we have subjected the samples to Fourier transform infrared spectroscopy analysis. Figure 3A shows the FTIR spectra of deep red colour aggregated sample (Oleylamine–Acetone aged sample) and for the comparison purpose the FTIR spectrum from Oleylamine and Acetone are also given. FTIR spectra for pure Oleylamine solution shows dominant sharp band at 2922 cm^−1^ and 2852 cm^−1^ corresponding to methyl asymmetric stretching from the terminal CH_3_ groups and methyl asymmetric C–H stretching from the CH_2_ groups in Oleylamine chain. The broad band at 1629 cm^−1^ corresponds to N–H bending and NH_2_ scissoring combined motions. FTIR modes in between 650–900 cm^−1^ corresponds to N–H wagging. The bands at 909, 964 and 993 cm^−1^ corresponds to NH_2_ bending mode. FTIR spectra for Acetone solution shows dominant mode at 1712 cm^−1^ corresponds C=O stretching mode. The other vibrational modes at 1426 and 1361 cm^−1^ corresponds to CH_3_ asymmetric and symmetric deformation. CCC asymmetric stretching mode of Acetone is at 1222 cm^−1^. The modes at 904 and 1091 cm^−1^ corresponds to CH_2_ rock vibrations. Oleylamine–Acetone aged samples show both vibrational modes from Oleylamine and Acetone molecules. C=O stretching mode of Oleylamine–Acetone aged samples red shifted to 1706 cm^−1^ compared to that of Acetone (1712 cm^−1^) as shown in Fig. 3B and that could be because of the formation of NH…O=C type hydrogen bond^48^ between hydrogen in the Oleylamine^49,50^ and oxygen in the Acetone. Figure 3C Schematic diagram illustrating the formation of hydrogen bond interaction between the Oleylamine and Acetone.

Raman spectroscopy analysis was used to confirm the formation of molecular clusters/aggregates. Figure S1 shows the Raman spectra recorded from Oleylamine–Acetone sample at the time of mixing and aged Oleylamine–Acetone molecular aggregate samples. For comparison purpose we have also recorded the Raman spectra from Oleylamine and Acetone samples as well^51,52^. The Oleylamine–Acetone sample at the time of mixing has shown the respective samples Raman modes as in Figure S1. The tables (Table S1 and Table S2) given in the supporting information explains about the details of the Raman modes. We have not seen any noticeable changes in the Raman modes of Oleylamine and Acetone at the time of mixing. However in the aged samples we have obtained a broad Raman spectrum as shown in Fig. S1. This interesting observation clearly tells a fact that there is correlation between the formation of molecular clusters/aggregates and broadening of Raman modes. We believe that the discrete vibrational levels of monomer type molecules becomes a relatively dense packed energy levels due to the clustering of molecules and when Raman excitation/de-excitation occurs through these dense energy levels a broader Raman band is resulted. The observation of broad Raman modes can be therefore taken as a confirmation for the molecular cluster formation. Further in order to know about the crystalline nature of the sample X-ray diffraction analysis was carried out from the Oleylamine as well as Oleylamine–Acetone aged sample by coating it as thin film on borosilicate glass substrate. We have obtained a broad diffraction peak at 22° corresponding to the 2θ of amorphous carbon^53^ (Fig. S2) and it indicated the non-crystallinity of the molecular cluster/aggregates.

**Figure 4:**
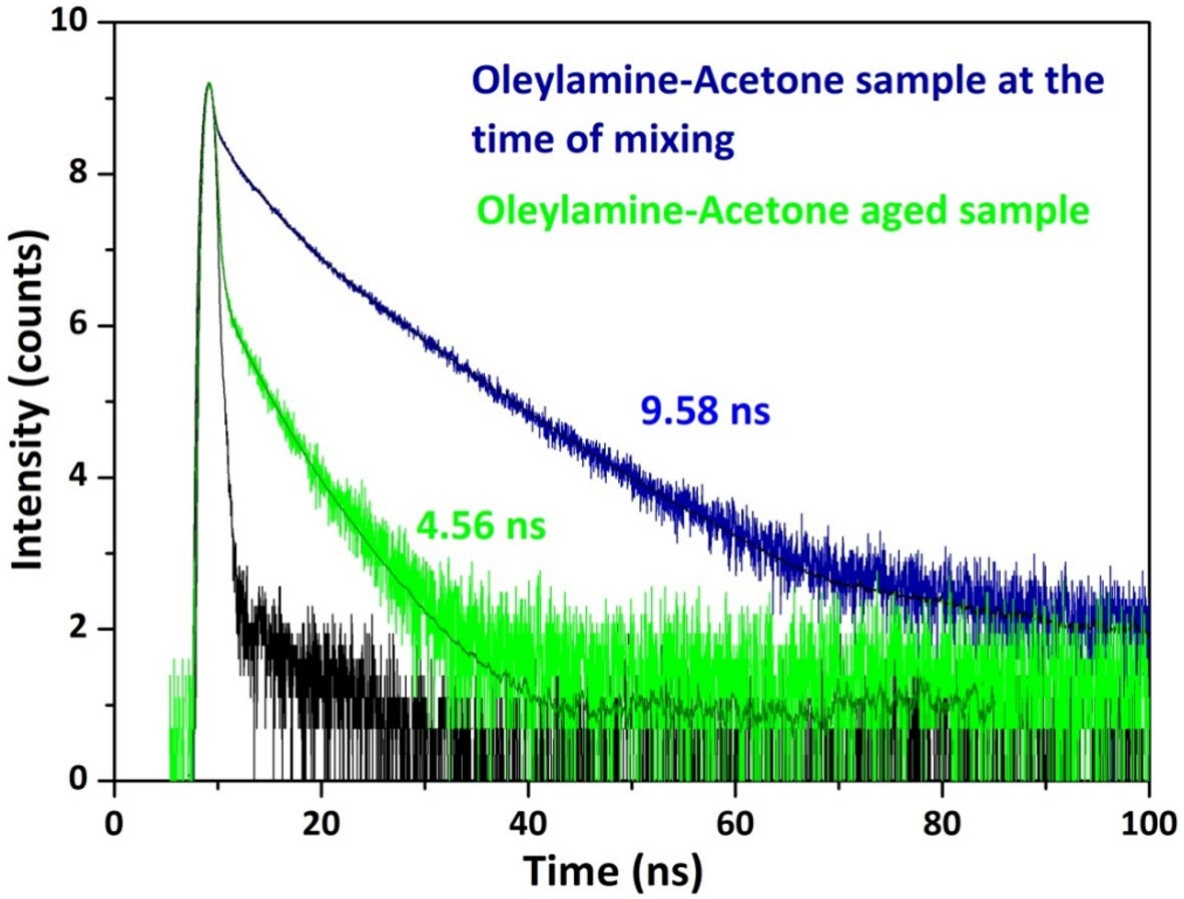
Lifetime decay curves denoting the excited state carrier lifetimes of Oleylamine–Acetone system at the time of mixing and after ageing. The lesser average lifetime value of the aged sample is an indication that the photo excited charge carriers of molecular aggregate system are undergoing a rapid relaxation through the coupled clusters.

Time correlated single photon counting technique was used to find the excited state lifetime of as-prepared samples, which is shown in Fig. 4. Oleylamine–Acetone sample at the time of mixing exhibited an average lifetime value of 9.58 ns. After the aging process for 1 month the sample exhibited lower average lifetime value of 4.56 ns compared to initial day sample. The observed results indicated a fact that the photoexcited charge carriers are undergoing a rapid relaxation through the coupled aggregates resulting of decrease in the average lifetime value in the case of aged sample. We have also repeated the experiment with different ratio of solute (Oleylamine)/solvent (Acetone). The solute:solvent ratio taken are 5 ml: 0.5 ml, 5 ml: 1 ml, 5 ml: 1.5 ml, 5 ml: 2 ml, 5 ml: 2.5 ml, 5 ml: 3 ml, 5 ml: 4 ml, 5 ml: 5 ml, 5 ml: 6 ml, 5 ml: 7 ml, 5 ml: 8 ml, 5 ml: 9 ml and 5 ml: 10 ml. The mixed solutions were kept under aging for 30 days. When 0.5 ml of Acetone is mixed with 5 ml Oleylamine, then we have noticed blue emission even after aging for several days. However, when 1 ml Acetone is mixed with Oleylamine of 5 ml, an interesting observation is noticed. Now the aged solution did not turn completely into red. Instead the top portion of the container has turned into orange, leaving the bottom portion as transparent solution (Immiscible solution). When we have slowly increased the Acetone volume to 2 ml, 3 ml, 4 ml, 5 ml and 6 ml then there we have noticed a change in the ratio between the orange solution to the transparent solution, upon aging. In particular the orange portion of the solution (volume) was increasing with the increase of Acetone addition. When 7 ml, 8 ml, 9 ml and 10 ml Acetone were added then the entire solution was turned into orange to deep red solution upon aging (Miscible solution). The critical quantity of Acetone required to turn the entire solution to red colour (upon aging) was found to be 7 ml with 5 ml Oleylamine.

When the samples having two regions were seen through UV light excitation, interestingly we have noticed two different emission colours as shown in Fig. S4. The top portion has slowly turned from blue to yellow colour after one month and yellow to orange colour after two months, whereas the bottom portion remained blue in colour always. To understand the nature of the solution in two different regions, FTIR spectrum was taken for both portions of the samples (Fig. S4). The aged bottom portion has shown the signature for the presence of Water and Acetone. The presence of Water indicated a fact that there is a possibility for the condensation reaction between Oleylamine and Acetone leading to the formation of Oleylimine. Further from FTIR analysis of the bottom portion of sample we have noticed hydrogen bond interaction between Water molecule and Acetone and that reflected with a redshift in vibrational mode of C=O stretching to 1694 cm^−1^^54^. The top portion has shown the signature for the presence of both imine and hydrogen bonded Oleylamine–Acetone molecules. The FTIR modes at 1662 cm^−1^ corresponds to C=N stretching and 1710 cm^−1^ corresponds to hydrogen bonded C=O stretching were the evidences for the presence of both Oleylimine and hydrogen bonded Oleylamine–Acetone clusters^55^, respectively. The Oleylimine molecule (imine bonded Oleylamine–Acetone molecular structure) formed at the bottom of the beaker during the condensation reaction reaches the top portion of the solution and become responsible for the FTIR mode at 1662 cm^−1^. Thus from overall experimental observations we understand the following facts. At a critical ratio between the solute and solvent, Oleylimine formation is not effective, instead hydrogen bond mediated Oleylamine–Acetone molecular clusters and aggregates are preferentially formed. It means that when we are taking the solute and solvent at the relative critical concentration then hydrogen bond formation favorably taking place.

**Figure 5:**
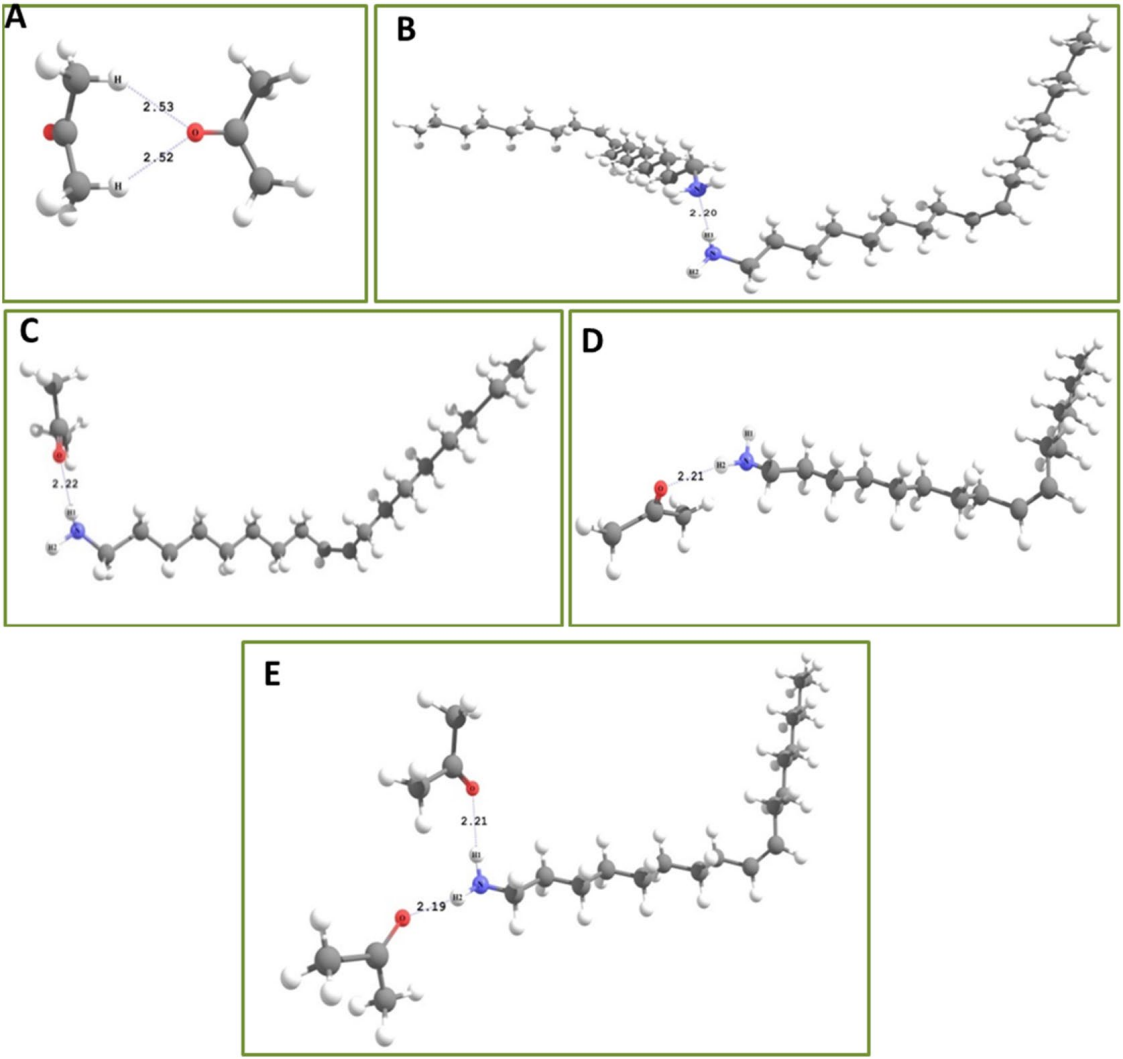
Optimized geometry of studied molecular complexes; (**A**) Acetone interacting with another Acetone, (**B**) Oleylamine interacting with another Oleylamine, (**C**) Acetone interacting with hydrogen (H1) of Oleylamine, (**D**) Acetone interacting with hydrogen (H2) of Oleylamine. (**E**) Two Acetones interacting with two hydrogen’s of Oleylamine.

**Figure 6:**
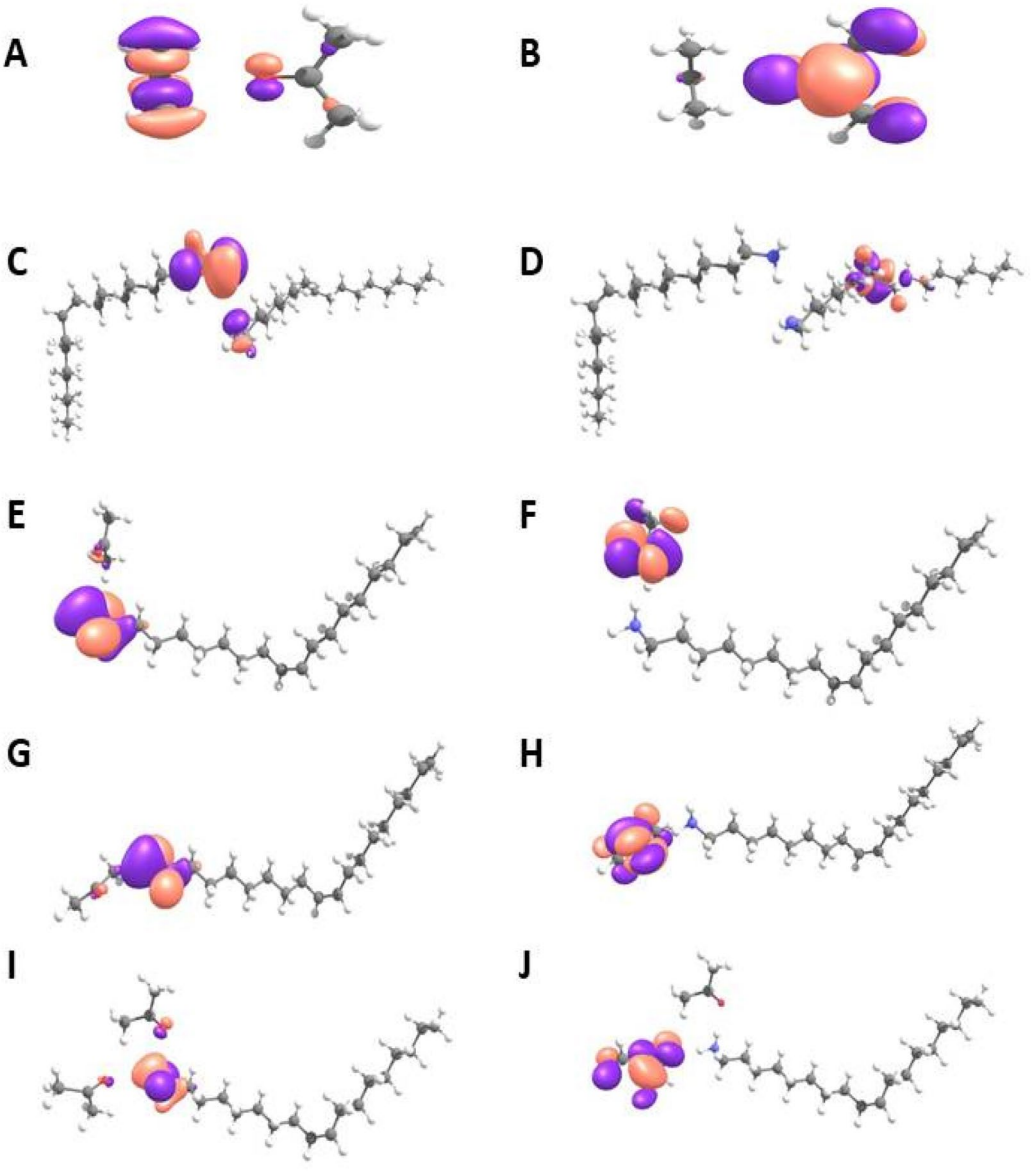
Representation of HOMO–LUMO plots in studied complexes (**A**) HOMO for Acetone- Acetone complex, (**B**) LUMO for Acetone–Acetone complex, (**C**) HOMO for Oleylamine–Oleylamine complex, (**D**) LUMO for Oleylamine–Oleylamine complex, (**E**) HOMO for Oleylamine (OLA1)–Acetone complex, (**F**) LUMO for Oleylamine (OLA1)–Acetone complex, (**G**) HOMO for Oleylamine (OLA2)–Acetone complex, (**H**) LUMO for Oleylamine (OLA2)–Acetone complex, (**I**) HOMO for Oleylamine (OLA12)–Acetone complex, (**J**) LUMO for Oleylamine (OLA12)–Acetone complex.

Theoretical work also has been conducted in order to understand the formation of hydrogen bond and its stability in Oleylamine–Acetone molecular system, through quantum chemical calculations. Theoretical emission spectrum of Oleylamine were obtained in Air and Acetone solvent medium and this is to understand the environmental effect on Oleylamine emission spectra. Figure S5 is the simulated emission spectra of Oleylamine in gas medium and Oleylamine in Acetone solvent medium, intended for the study of solvatochromic effects on Oleylamine in Acetone solvent. The spectral curve is clear indicative of solvatochromic effect leading to red shifting of Oleylamine molecule emission spectrum in Acetone solvent medium. In gas medium spectral distribution of Oleylamine seems to be in shorter UV range of wavelength, suggestive of its colourless property. Though Oleylamine shows well distinguishable red shifted spectral behaviour in Acetone solvent medium in the experimental case, its spectrum still in longer UV range in theoretical data. These observations are suggestive that though solvatochromism of Oleylamine in Acetone solvent red-shifts its gas phase spectra, solvatochromism alone could not explain the experimentally observed vast spectral changes occuring in Acetone solvent upon aging. Hence we speculate that the experimentally observed red shifted emission probably involves reasons beyond solvatochromism. In this case there is a possibility for hydrogen bond interaction between solute–solvent molecules. Since Oleylamine has amine (–NH_2_) ending and Acetone has open oxygen atom (–C=O), hydrogen bonding could possibly happen between those molecules. Possibilities of other non-covalent interactions in other sites of molecules are seems to be low. Hence we carried out quantum chemical analysis on probable hydrogen bonding sites where electronegative atoms and hydrogen atoms are co-existent. Figure 5. represents optimized geometries of interacted Oleylamine–Acetone complexes of various combinations. It is important to note that stronger interactions are characterised by shorter bond length and larger interaction energy. Table 1. enumerates interaction energies of each of these complexes. From bond length and interaction energy it could be observed that Ace_Ace (Acetone interacting with another Acetone) interaction is the weakest among all interactions, OLA_OLA (Oleylamine interacting with another Oleylamine) interaction, OLA1_Ace (Hydrogen H1 in the Oleylamine interact with one Acetone) and OLA2_Ace (Hydrogen H2 in the Oleylamine interact with one Acetone) possess comparable interaction strength, while OL12_Ace ( Hydrogens H1 and H2 in the Oleylamine interacts with two Acetones) shows strongest interaction among the studied complexes. When solute–solute interaction and solvent–solvent interaction are stronger than solute–solvent interaction, one would observe solvophobic effect that results in immiscible mixture of solute and solvent. However from the obtained results we could see that solution–solution interaction (Ace-Ace) is clearly weaker than solute–solvent interactions (OLA1_Ace, OLA2_Ace and OL12_Ace). Further, though solute–solute (OLA_OLA) shows stronger interaction than solute–solvent interactions (OLA1_Ace, OLA2_Ace and OL12_Ace), in terms of bond distance, and vice-versa in terms of interaction energy based values we see solute–solvent and solute–solute interactions are close in interaction strength. Therefore, we conclude that the discussed mixture might show partial solvophobic effect, where Acetone solvent will try to penetrate Oleylamine solute to form hydrogen bonding with Oleylamine molecules. On the other hand, hydrogen bonding interactions of Oleylamine molecules with another Oleylamine will try to mitigate the hydrogen bonding interaction between Acetone molecules and Oleyamine, as a result we assume a persistent competition of interactions between solute–solvent and solute–solute species will be there leading to the formation miscelle type configuration. Figure 6 shows the HOMO–LUMO distributions observed in the studied complexes, which clearly shows that HOMO of Oleylamine and LUMO of Acetone are crucial orbitals involved at hydrogen bonding sites. HOMO and LUMO distributions in solute–solvent interacted complexes shows localization of HOMO on Oleylamine and localization of LUMO on Acetone, which is indicative of charge transfer type excitations in these systems (donor–acceptor kind of complex). Such complexes are usually stabilized by electrostatic attractions. Among all the studied complexes, OL12 shows much reduced HOMO–LUMO gap (Table S3). The relative energy level position for all the studied complexes are also given in Fig. S6. Therefore when such molecular clusters with reduced HOMO–LUMO gap are formed then there is possibility for the red shifted emission, assisted by both the reduction of HOMO–LUMO gap and delocalization effect through cluster. By comparing the experimental observations and theoretical results, we arrive to a conclusion that the possibility to form imine bond mediated Oleylamine–Acetone molecular network and hydrogen bond mediated Oleylamine–Acetone molecular clusters are both possible. However their dominant depends on the relative solute–solvent ratio. Development of hydrogen bond between Oleylamine and Acetone leads to the formation of a hetero structure type molecular network and when these hetero structure molecular system comes into contact in a head to tail pattern, a molecular clustering process is initiated thus leading to the formation of a nanoscale sized molecular clusters, at first, and then their self-assembly leading to the formation of flower like aggregation later. The nanoscale clustering cum aggregation process occurs in all parts of the mixed solution when the solute–solvent ratio reaches critical value. If there is a decrease in the solution (Acetone) content then two different types of reactions occur in the solute–solvent container. In one type of reactions hydrogen bond is established between Oleylamine and Acetone leading to the formation of flower like aggregates as discussed before. In the other type of reaction covalent interaction is established between Oleylamine–Acetone molecules leading to the formation Oleylimine molecules and Water as by products. The experimental observations show the sensitiveness of the ratio between the solute and solvent in deciding the type of reactions between the molecules. As we observe the formation of molecular aggregates at a critical solute–solvent ratio, wherein the solution (Acetone) concentration is higher, we believe that Oleylamine (solute) will be surrounded by Acetone (solution) resulting into a micelle structure and in this pattern hydrogen bonding is favoured between the Oleylamine and Acetone molecules, which finally leads to the formation of nanoscale molecular clusters and flower like aggregates. When there is no enough coverage to surround solute molecules, such micelle type structures are not seems to be formed and therefore hydrogen bond type interaction between the molecules is not favoured. The excess solute therefore settles at the bottom as a separate phase, thus producing two different regions in the sample. The formation of an interface between two liquid phases could be the driving force of condensation reaction between Oleylamine and Acetone. Interface assisted (Immiscible liquids) polymerization^56^, liquid–air interface assisted peptide bond formation^57^ are the best examples for the conversion of monomers into a big molecule. At the interface when monomer molecules try to diffuse from one region to other there is a high possibility to have interaction between them and therefore monomer becomes large sized molecules. Here we believe that when monomer molecules (Oleylamine and Acetone) diffuse from one part of the liquid to other there is a close interaction between them leading to the formation of Oleylimine, leaving Water molecules as byproduct. Therefore from overall experimental/theoretical studies we come to a conclusion that the hydrogen bond type chemical interaction between Acetone and Oleylamine is due to the formation of micelle structure and relative stronger solute–solvent and solute–solute interaction between the molecules. On the other hand Oleylimine is formed due to the interfacial effect and that happens at a different ratio between solute–solvent mixing. The photographic image of of different dilutions of Oleylamine–Acetone aged samples under UV laser illumination as shown in Fig. S7.

**Table 1:** Interaction energies of solute (Oleylamine) and solvent (Acetone) calculated at sB3LYP/6-311G (d,p) level of theory with BSSE corrections. (i) Acetone-Acetone complex, (ii) Oleylamine–Oleylamine complex, (iii) Oleylamine (OLA1)–Acetone complex, (iv) Oleylamine (OLA2)–Acetone complex, (v) Oleylamine (OLA12)–Acetone complex.

Complex	Interaction energy (kcal/mol)
Ace_Ace	− 2.04
OLA_OLA	− 2.76
OLA1_ACE	− 2.8
OLA2_ACE	− 2.98
OLA12_ACE	− 3.06

**Figure 7:**
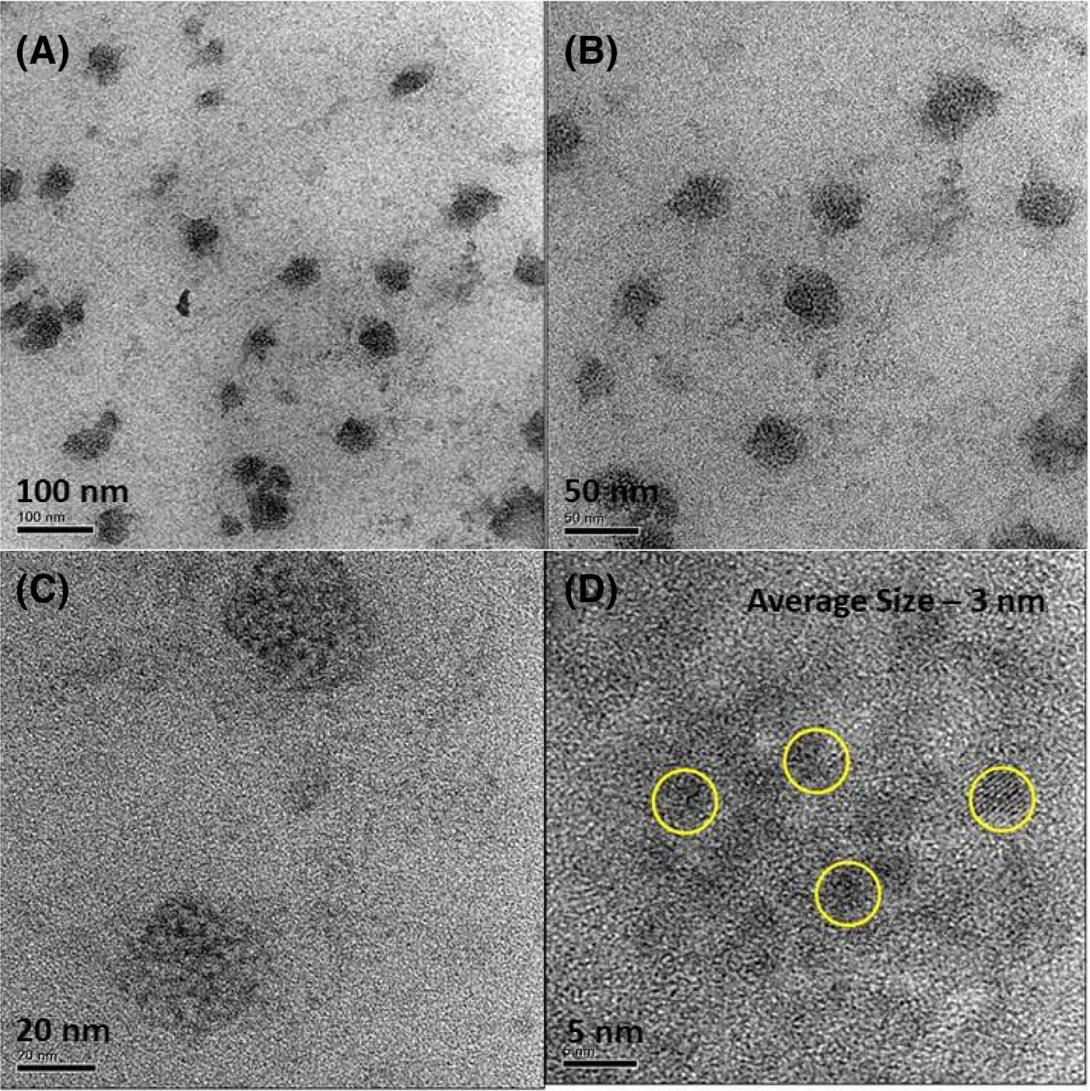
(**A**–**C**) HRTEM images of In_2_S_3_ QD system before aging process at different magnifications, showing a cluster type QD arrangement in the as prepared sample. Quantum dots exhibited an average size of 3 nm.

**Figure 8:**
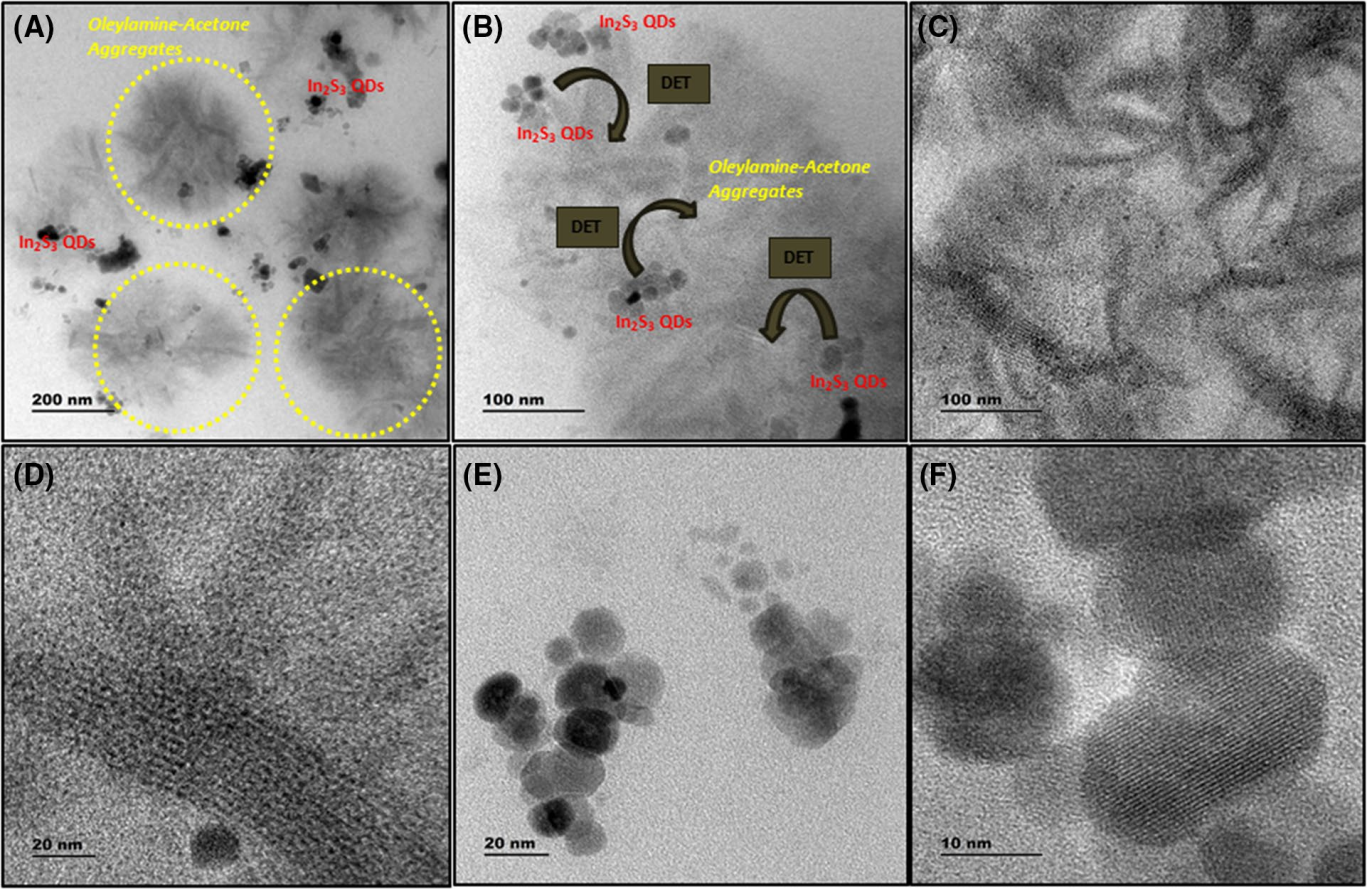
(**A**–**C**) shows the HRTEM images of aged In_2_S_3_ QD sample with larger sized In_2_S_3_ QD embedded in the matrix of molecular aggregates. Yellow circles indicate the highlighted portions of the image showing the presence of molecular aggregates (**A**). In_2_S_3_ quantum dots embedded in the Oleylamine–Acetone molecular aggregates is clearly seen in (**B**). (**D**) is the High resolution image of the molecular aggregates present in the In_2_S_3_ QD sample and which is similar to the HRTEM image as seen before in the molecular system. (**E**,**F**) are the HRTEM images of In_2_S_3_ QD showing an average size of 19 nm. The presence of molecular aggregates in the In_2_S_3_ QD system indicated a fact that the aging assisted process has removed the Oleylamine from QD surface and hence the size of the quantum dot gets increased after aging process.

Since Oleylamine is an important surfactant molecule used to prepare size controlled quantum dots, Acetone assisted aging process was done in In_2_S_3_ quantum dot samples, as an example system. First we have prepared Oleylamine and 1-Dodecanthiol capped blue emitting In_2_S_3_ QDs by Hot Injection Technique (Details are given in the experimental part). Then the QDs were dissolved in an optimized Acetone concentration and kept under room temperature aging process for one month. Figure 7 shows the HRTEM images of as prepared In_2_S_3_ QD system, which shows the clustered type QDs with an average size of 3 nm. In contrast to this as prepared sample’s HRTEM image, the aged sample’s HRTEM image has shown a different morphology, consisting of In_2_S_3_ QDs with relatively larger size (average size: 19 nm) and as well as Oleylamine–Acetone molecular clusters, as we have seen previously. The size distribution corresponding to larger sized In_2_S_3_ QD is given in the supplementary information. In_2_S_3_ QDs were found to embedded in the Oleylamine–Acetone molecular aggregate matrix as shown in Fig. 8. The carbonyl group in the Acetone molecules formed hydrogen bond interaction with amine group in the Oleylamine attached to the In_2_S_3_ QDs and that leads to the detachment of Oleylamine molecules from QD surface and as a consequence there is an increase in QD size. In the absence of sufficient surface coverage smaller sized In_2_S_3_ QDs started to grow into larger sized QD system is the reason for this observation. The optical absorption and emission spectra were recorded from the aged sample and compared it with the data of as prepared samples. Interestingly we have seen a shift in the emission peak position from blue to red wavelength, as noticed previously in the molecular aggregate samples. This shift could be due to the possible energy transfer interaction between blue emission giving In_2_S_3_ QD and red emitting Oleylamine–Acetone cluster system. As the broad absorption band position of the molecular cluster has overlapped with the emission maximum of blue emitting In_2_S_3_ QD, there is a high possibility to have energy transfer interaction between In_2_S_3_ QD and molecular cluster system. The presence of In_2_S_3_ QD in the matrix of molecular cluster thus enable the transfer of photoexcited charge carriers from In_2_S_3_ QD to molecular system. To use this advantage in the photo current generation, a photo current device was constructed using the In_2_S_3_ QD/Oleylamine–Acetone molecular aggregate hybrid system as an active layer. For comparison purpose devices were also constructed with In_2_S_3_ QD free molecular cluster sample as an active layer as well.

**Figure 9:**
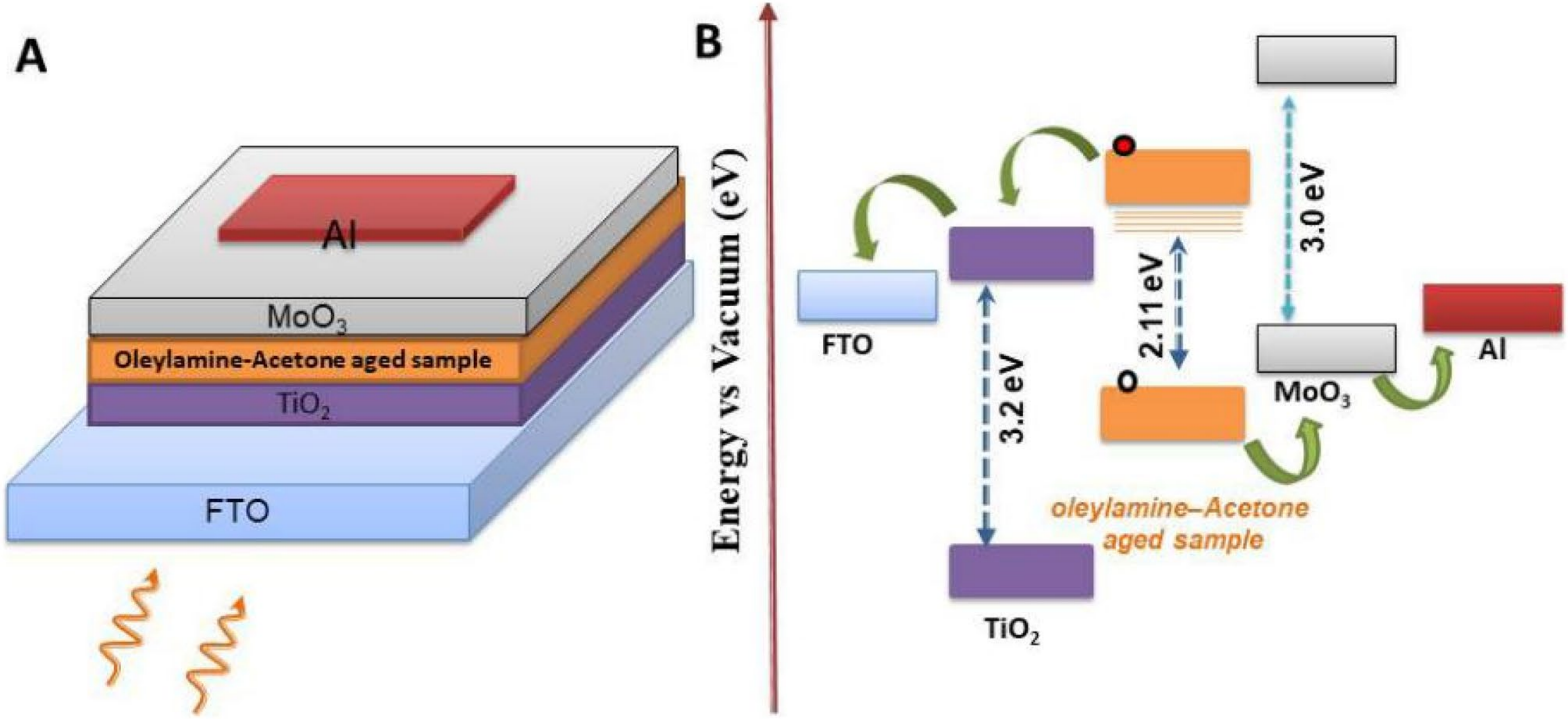
(**A**) Photocurrent device structure and (**B**) the possible relative energy band diagram for the Oleylamine–Acetone aged sample as an active layer with following structure FTO/TiO_2_/Oleylamine–Acetone aged sample (molecular aggregates)/MoO_3_/Al.

**Figure 10:**
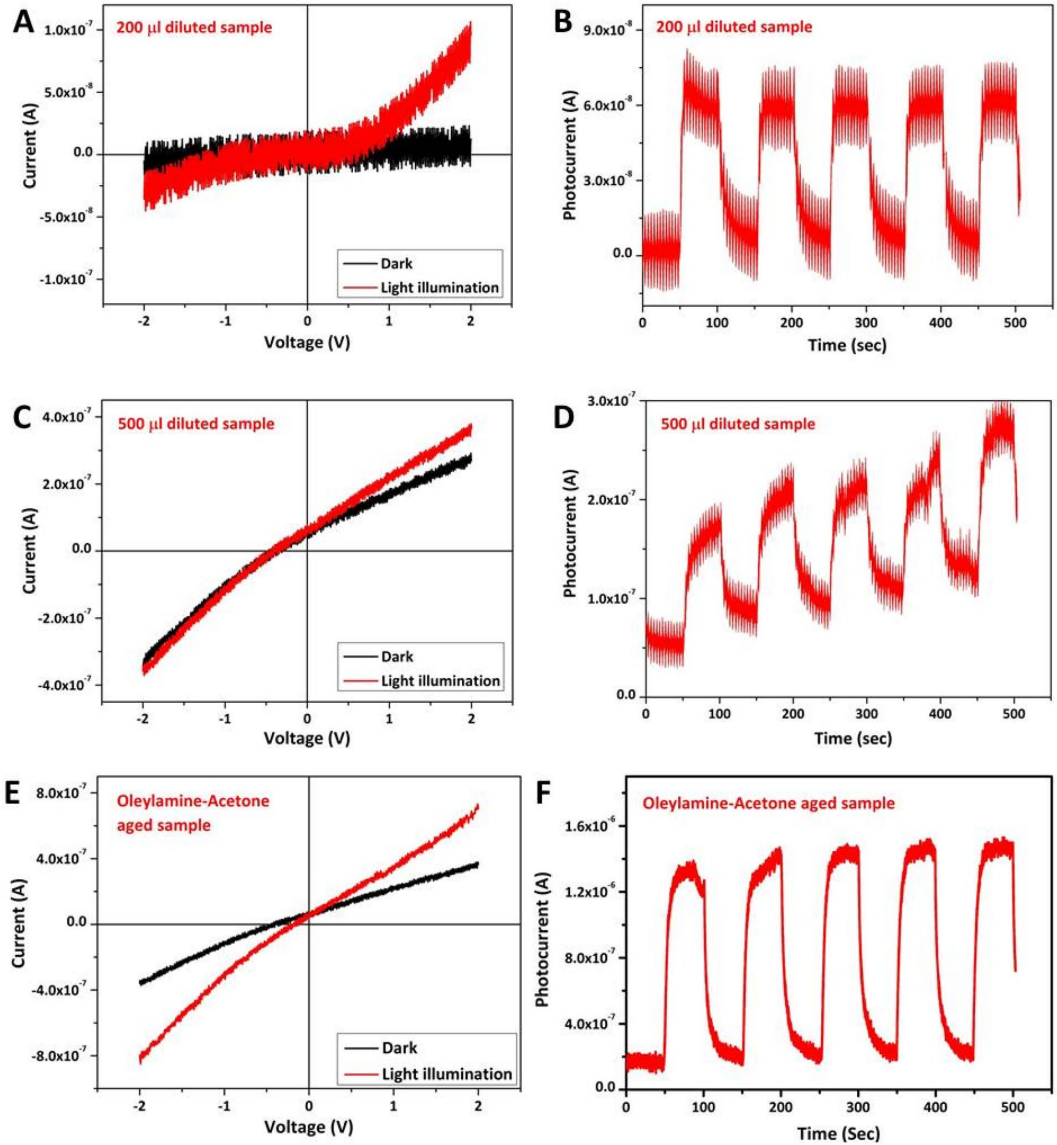
Current (I) (vs) Voltage (V) characteristics curves and Photocurrent (I) (vs) Time (t) plots from different samples when incorporated into the above device structure. (**A**,**B**) Corresponds to 200 μl diluted sample’s I vs V and I vs t plots. (**C**,**D**) corresponds to 500 μl diluted sample’s plots. (**E**,**F**) corresponds to as prepared aged samples plots. Among different samples, the aged molecular aggregates produced higher photocurrent compared to other diluted samples and that could be associated with an effective delocalization of charge carriers through coupled clusters.

**Figure 11:**
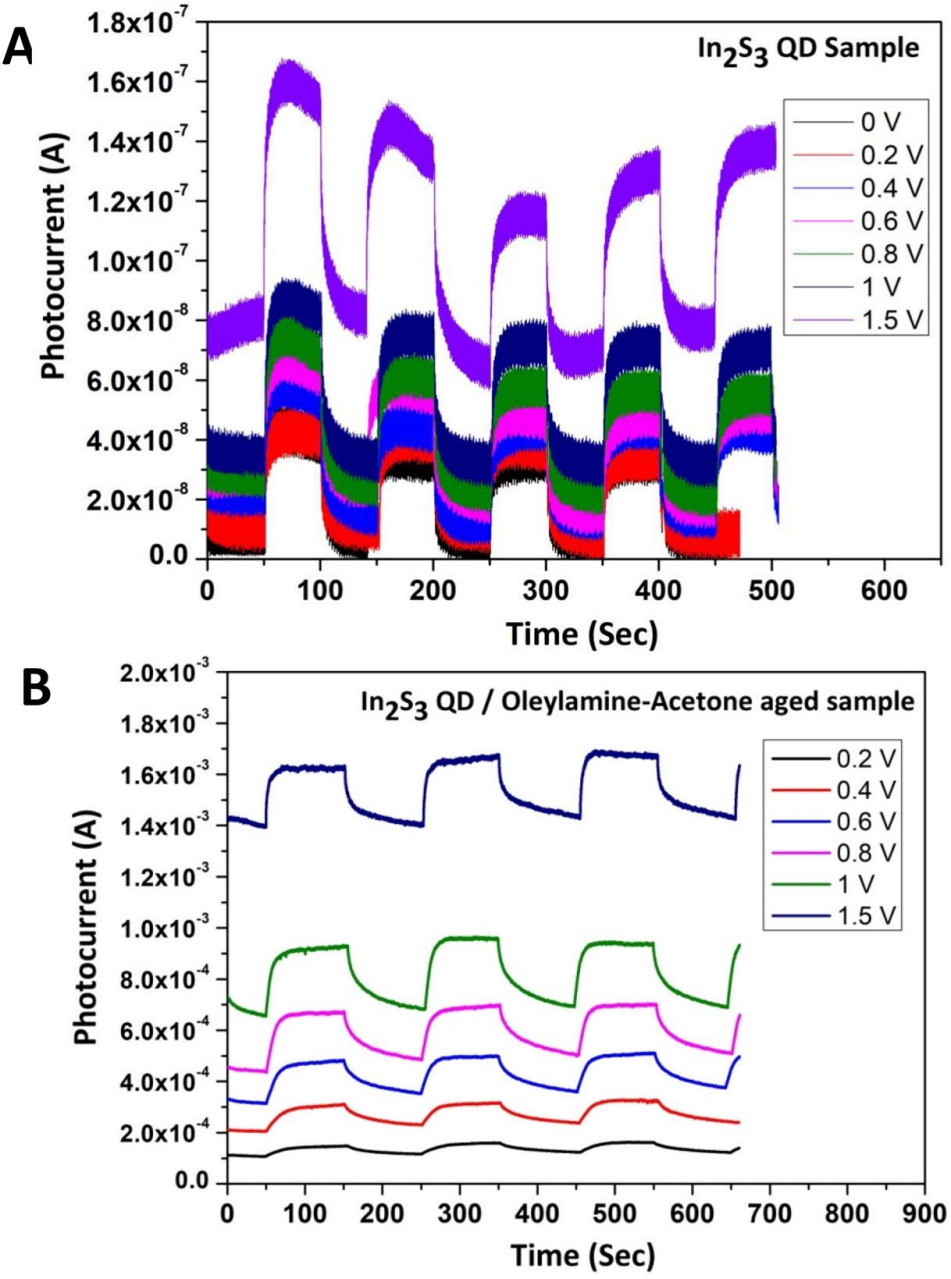
(**A**) Photocurrent (I) vs Time (t) plot of as prepared In_2_S_3_ QD sample at 50 s ON/OFF Ratio with different biasing voltages (**B**) Photocurrent (I) vs Time (t) plot of aged sample consisting of In_2_S_3_ QD/Oleylamine–Acetone aggregates at 100 s ON/OFF Ratio with different biasing voltages.

The structure of the photocurrent device fabricated using the as formed cluster solutions as photoactive material and the relative energy level diagram are shown in (Fig. 9). The device constructed using Oleylamine–Acetone sample at the time of mixing did not produce any photo current upon illumination with solar simulator. On the other hand device constructed using Oleylamine–Acetone aged sample shows good photocurrent value of 1.3 µA at 50 s ON/OFF ratio time with the biasing voltage of 1 V (Fig. 10). The I–V curves of the samples were also recorded at dark as well as light illumination condition. There is a considerable increment in the photocurrent value after light illumination compared to the dark condition reveals that the molecular clusters are sensitive to incident light and able to produce photocurrent. It has to be noted that non-zero dark current in the samples could be due to leakage of charges through the defect levels present in the samples. Since the active and other supportive layers are sandwiched between two electrodes with different work functions, a potential drop will be there across the device structure to drive the trapped charge carriers through the defect levels under dark condition resulting into a non-zero dark current at zero applied bias. The magnitude of this dark current shall follow a linear trend when external bias is applied. Photo current device fabricated with aged sample can produce high magnitude photocurrent because exciton delocalization through self assembled aggregates is much easier. On the other hand the photo current device fabricated with diluted samples shows relatively lesser magnitude photo current, and that can be related to dissociation of self-assemblies which can decrease the exciton delocalization. The photo current values of 0.16 μA and 0.06 μA were obtained from the photo current devices made up of 500 μl and 200 μl diluted samples. In the device, if the In_2_S_3_ QD aged sample was used as an active material then we have noticed a high magnitude photocurrent in mA range as shown in Fig. 11B. For comparison purpose In_2_S_3_ QD un-aged samples photocurrent data are also given in Fig. 11A, and which shows lesser magnitude photocurrent. The presence of In_2_S_3_ QD in the matrix of Oleylamine–Acetone aggregates pumps in more photo generated charge carriers into the molecular cluster system and therefore a high magnitude photocurrent is possible in the hybrid sample. Since the as prepared aggregates are J-type with head to tail dipole arrangement and as this arrangement is helping to delocalize the charge carriers, the photo excited charge carriers transferred from In_2_S_3_ QD can be easily conducted through the molecular aggregates, therefore, high magnitude photocurrent is possible. From the comparison of all of the photocurrent data, it is concluded that there is an overall increase in the photocurrent magnitude when aged In_2_S_3_ QD sample is used and which supported our claim that the photoexcited charges are transferred to molecular cluster system through energy transfer interaction. Since, most of preparation experiments of colloidal QDs are done by organic surfactants/ligands such as Oleylamine, Oleic acid and tri-octyl phosphine oxide, which are introduced during colloidal QDs synthetic procedures to control nucleation and growth, their presence in the device structure has some disadvantageous effect on the device performance. For example, these ligands on the colloidal QDs surface are usually electrically insulating and prohibits the hopping of photo induced excited charge carriers from one QD to other and as a result, a less number of photo excited charge carrier reaches to electrode. Hence therefore, the replacement of bulky surfactants are essential to increase the charge carrier transport within the colloidal QDs or from QDs to another nanostructured system (for eg: to TiO_2_ electrode) for an improved photocurrent generation. It is therefore various efforts have been taken to post synthesis replacement of capping ligands with short chain molecules or mono atomic ligands (Cd, S, Br or I) to form interconnected QD network structure. Here we have used a different approach to remove the surfactant molecule (Oleylamine) from In_2_S_3_ QD surface by aging assisted cluster formation method. Interestingly the as formed molecular aggregates are found to be acting as a conducting platform to transport the photo excited charge carriers from In_2_S_3_ QD system and enhanced photocurrent is generated. It is therefore believed that our approach can solve the problem associated with the removal of surfactant from QD system, in the future.

## Conclusions

In conclusion, through experimental and quantum chemical calculation results we have realized hydrogen bond mediated heterostructure type molecular cluster formation and their aggregation, resulting of red shift in the emission wavelength for the first time. Aging assisted formation of NH…O=C type hydrogen bond between Oleylamine and Acetone leads to the formation of nanoscale molecular clusters. Once the nanoscale molecular clusters were formed then the formation of flower like aggregates and their dissociation as well as reformation were rapidly occurred by means of controlling the dilution level of flower like aggregates in Acetone. Dissociation and reformation of flower like aggregates leads to the red to blue and blue to red shift in emission characteristics in a rapid manner. The sensitiveness of the ratio between solute/solvent in deciding covalent or non-covalent hydrogen bond type interactions were elaborated in Oleylamine–Acetone molecular system. Covalent interaction between Oleylamine and Acetone was possible due to the formation of liquid–liquid interface between molecular cluster and non-cluster solution, followed by diffusion of monomers through interface. Further high current giving photoconductive device was also realized from the molecular cluster/aggregate system. When this implication (molecular cluster formation) was introduced in a real quantum dot system, there we noticed an further increase in the photocurrent generation.

## Supplementary information

Supplementary Information.
