# Supplementary material for: Aggregation induced emission behavior in oleylamine acetone system and its application to get improved photocurrent from In2S3 quantum dots
Source: Sci Rep. 2020 Nov 12;10:19712. doi: 10.1038/s41598-020-76703-0 (PMC7661720; doi:10.1038/s41598-020-76703-0)
Supplement: Supplementary file 1 — Supplementary Information. [file 41598_2020_76703_MOESM1_ESM.doc]

**Supplementary Information**

**Hydrogen Bond Assisted Formation of Oleylamine Acetone Molecular Aggregates and Their Application to get Improved Photocurrent from In_2_S_3_ Quantum Dot System**

Subramaniam Ramya^1^, Devaraj Nataraj^1,2^*, Sangameswaran Krishnan^3^, Sellan Premkumar^1,4^, Thankappan Thrupthika^1^, Arumugam Sangeetha ^1^,Kittusamy Senthilkumar^3^, T. Daniel Thangadurai^5^

1. Quantum Materials & Devices Laboratory, Department of Physics, Bharathiar University, Coimbatore, Tamil Nadu 641046, India.
2. UGC-CPEPA Centre for Advanced Studies in Physics for the development of Solar Energy Materials and Devices, Department of Physics, Bharathiar University, Coimbatore, Tamil Nadu 641046, India.
3. Molecular Quantum Mechanics Laboratory, Department of Physics, Bharathiar University, Coimbatore, Tamil Nadu 641046, India.
4. School of Chemistry and Chemical Engineering, Tiangong University, Tianjin 300387, China, and School of Material Science and Engineering, Tiangong University, Tianjin 300387, China.
5. Department of Nanoscience and Technology, Sri Ramakrishna Engineering College, Coimbatore, Tamil Nadu 641022, India.

Corresponding Author E-mail: [de.natraj2011@gmail.com](mailto:de.natraj2011@gmail.com)

**Photocurrent device fabrication**

FTO substrate purchased from sigma Aldrich was cleaned using standard procedure. Then TiO_2_ paste was deposited on the FTO substrate over an area of 0.5 x 0.5 cm^2^ by using doctor blade technique. Then the coated substrate was subjected to annealing process at 450 °C for 12 hours in a muffle furnace. Then the sensitizer sample (molecular cluster or Quantum dot solutions) was coated on TiO_2_ layer by using spin coating method with 500 rpm speed of 500 rpm for 20 sec by taking similar volume of the sample. Then the sample is dried for one day in vacuum desiccator. After that thermal evaporation technique was used to deposit MoO_3_ layer on sensitizer surface. Then finally aluminium metal contact was made by using thermal evaporation method. Then top and bottom contacts were taken by using copper leads with the help of silver paste. The device was then exposed to photocurrent characterization.

**Characterization**

UV-Vis absorption studies for all the molecular solutions were carried out using Agilent CARY 60 spectrophotometer. Photoluminescence emission spectra were obtained using Horiba Jobin Yvon Fluoromax-4 Spectrofluorimeter. HRTEM characterization was carried out by JEOL JEM-2100 at an operating voltage of 200 kV. In the case of HRTEM analysis the samples with different dilutions were coated on copper grid with 100 meshes. FTIR characterization was done by Bruker Tensor27 (ATR mode) instrument. Raman analysis was carried out using LabRAM-HR Raman spectrometer (Horiba) with 532 nm laser excitation. Raman data were collected from liquid samples. The excited state lifetime of all the molecular sample solutions was analysed through IBH time-correlated single photon counting (TCSPC) system with the excitation at 390 nm. X-Ray diffraction measurements were carried out with an Xpert PRO PANalytical instrument. In the case of XRD analysis the molecular solutions were drop casted on borosilicate glass substrates. Photocurrent studies were carried out by using solar simulator having 300 Watt Xenon lamp as an illumination source and a Source Measuring Unit (Agilent B2912A).

**Computational details**

All the calculations were carried out using G09 package. Ground state optimizations of all the reported molecules and complexes were carried out at B3LYP/6-311G(d,p) level of theory. Further, the computation of HOMO-LUMO distribution and interaction energies were also done at B3LYP/6-311G(d,p) level of theory. Absorption spectra of chosen set of species of molecules were computed by TD-DFT calculations on ground state optimized geometries at CAM-B3LYP/6-31G(d,p) level of theory. While, the emission spectrum of chosen set of molecules were computed after excited state optimizations at CIS/6-31G(d,p), followed by TD-DFT calculation at CAM-B3LYP/6-311G(d,p) level of theory. The solvent effects were taken for consideration in while computing all the molecules and complexes using Polarizable Continuum Model (PCM); in addition, absorption and emission spectrum of Oleylamine were computed at gas phase for the sake of analysing solvatochromic effects.

**Raman Analysis**

**
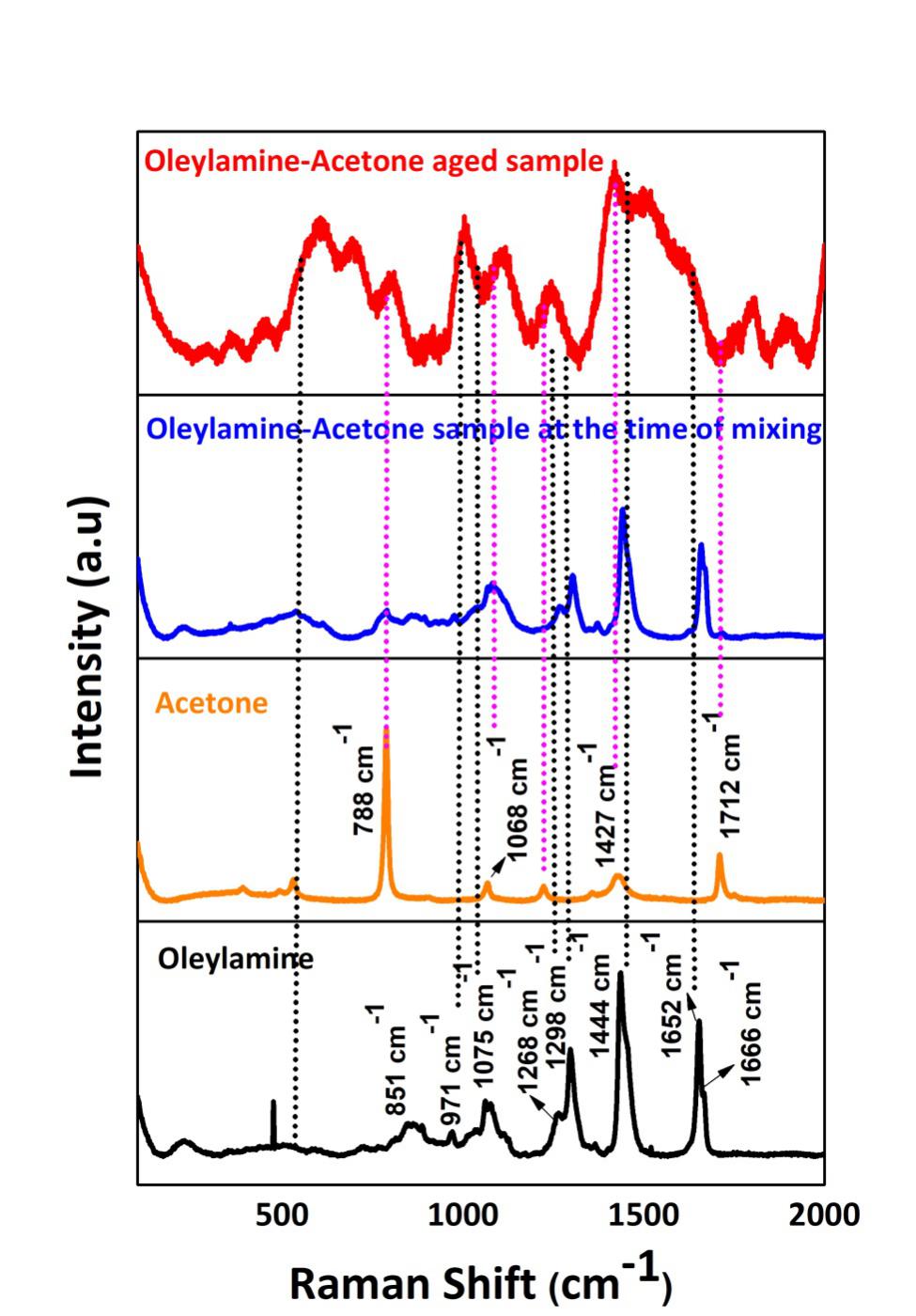
**

**Fig. S1** Raman spectra for Oleylamine, Acetone, Oleylamine-Acetone sample at the time of mixing and Oleylamine-Acetone aged sample. In the case of aged sample we have obtained relatively broader Raman modes from the Oleylamine-Acetone molecular aggregates.

**Raman Modes**

**Table S1. Raman modes of Oleylamine**

| **S. No** | **Raman modes (cm^-1^)** | **Assignment** |
| --- | --- | --- |
| **1** | 1666 | Trans-dialkyl C=C |
| **2** | 1652 | Cis-dialkyl C=C |
| **3** | 1444 | CH_2_ scissor and asym.bend |
| **4** | 1298 | CH_2_ twist |
| **5** | 1268 | Cis CH sym Rock |
| **6** | 1075 | C-N stretch |
| **7** | 971 | Trans C-H wag |
| **8** | 851 | C-C stretch and R-NH_2_ wag |

**Table S2. Raman modes of Acetone**.

| S. No | Raman modes (cm^-1^) | Assignment |
| --- | --- | --- |
| 1 | 1715 | C=O stretch |
| 2 | 1427 | CH_3_ deformation |
| 3 | 1068 | CH_3_ Rock |
| 4 | 788 | CC_2_ symmetric stretch |

**XRD Analysis**

**
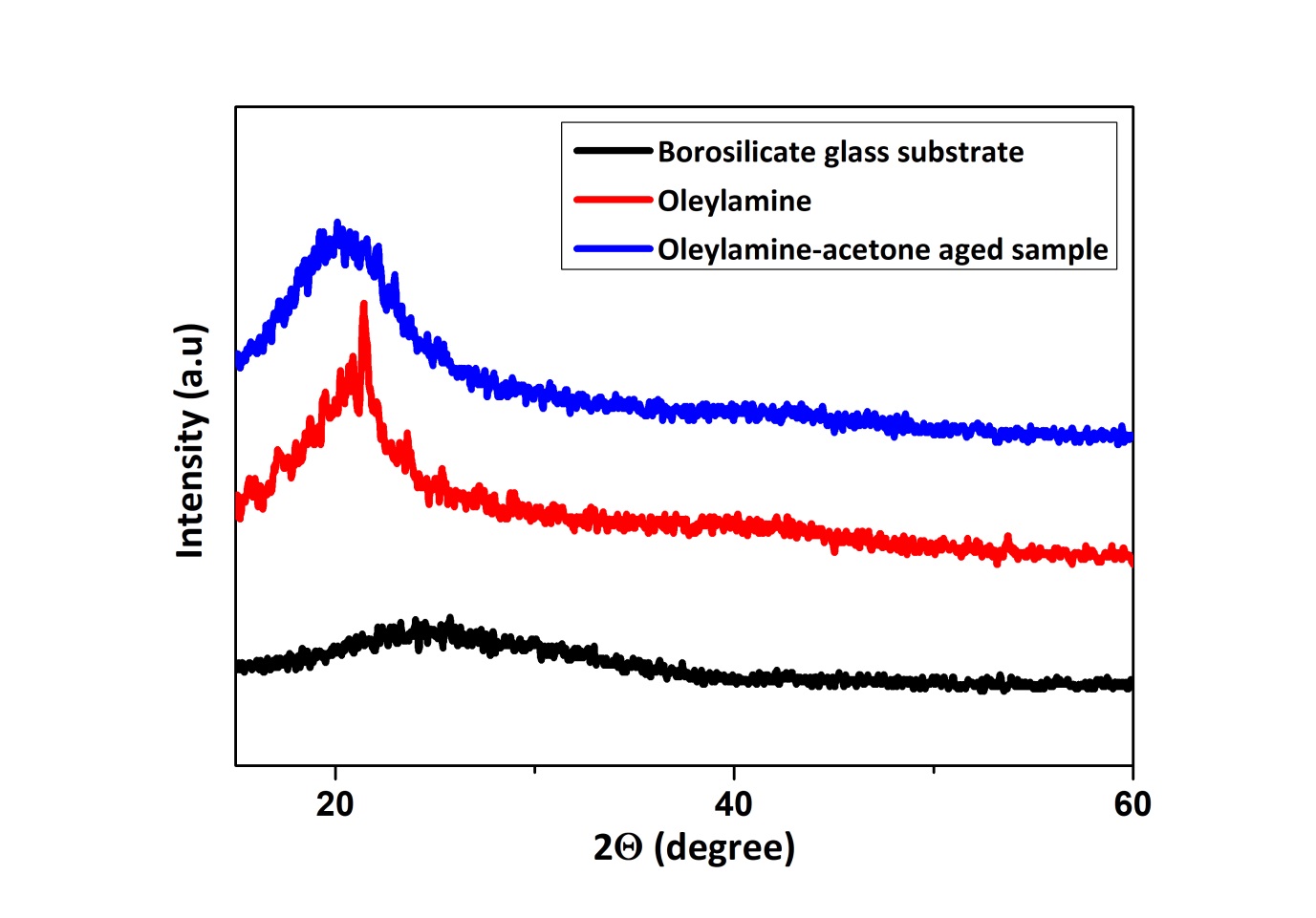
**

**Fig. S2** X-ray diffraction pattern for Oleylamine and Oleylamine-Acetone aged sample. A broader diffraction peak at 2θ value of 22^0^ corresponds to amorphous carbon and this indicates the non-crystalline nature of molecular cluster/aggregates.

**UV-Vis and Photoluminescence emission spectroscopy analysis for Amine-Acetone complexes**


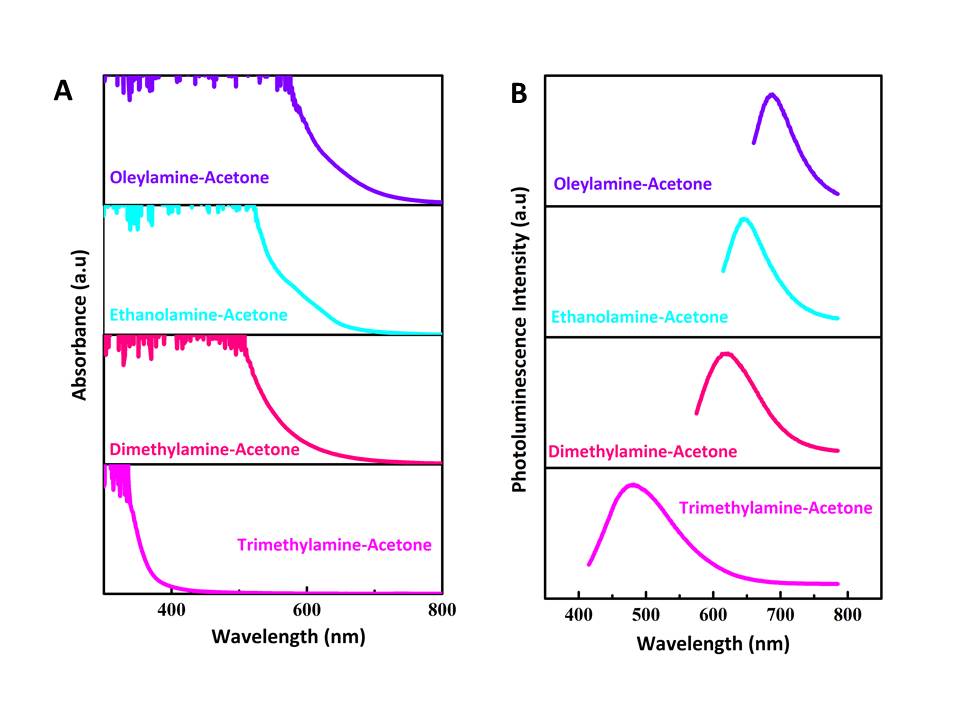


**Fig. S3** Absorption and emission spectra for different amines mixed with Acetone and kept under aging for 60 days. Primary amines (Oleylamine and Ethanolamine) exhibits more redshifted absorption and emission spectrum compared to that of Secondary and Tertiary amines due to the presence of two hydrogen atoms which preferably forms hydrogen bond with neighbouring Acetone molecules.

For comparison purpose we have taken different kinds of amines such as Ethanolamine (H_2_NCH_2_CH_2_OH), Dimethylamine ((CH_3_)_2_NH) and Trimethylamine ((CH_3_)_3_N) and adding them with Acetone, ageing assisted changes were observed. The corresponding samples absorption and emission spectra were recorded (**Figure S3**). Primary amines (Ethanolamine, Oleylamine) have shown a faster red shifted emission compared to Secondary amine (Dimethylamine). In the case of Tertiary amine (Trimethylamine) there is no such red shift. Primary amines can easily form hydrogen bonding with the neighbouring Acetone molecules because of two hydrogen atoms. But in the case of Secondary amines the ability of forming hydrogen bond with the neighbouring Acetone molecules gets decreased due the existence of one hydrogen atom and that has reflected in a slower red shift in the absorption and emission spectra of this sample. Tertiary amine could not form any hydrogen bonding interactions with the neighbouring Acetone molecules and hence it does not show any red shift in emission.

**FTIR Spectra**


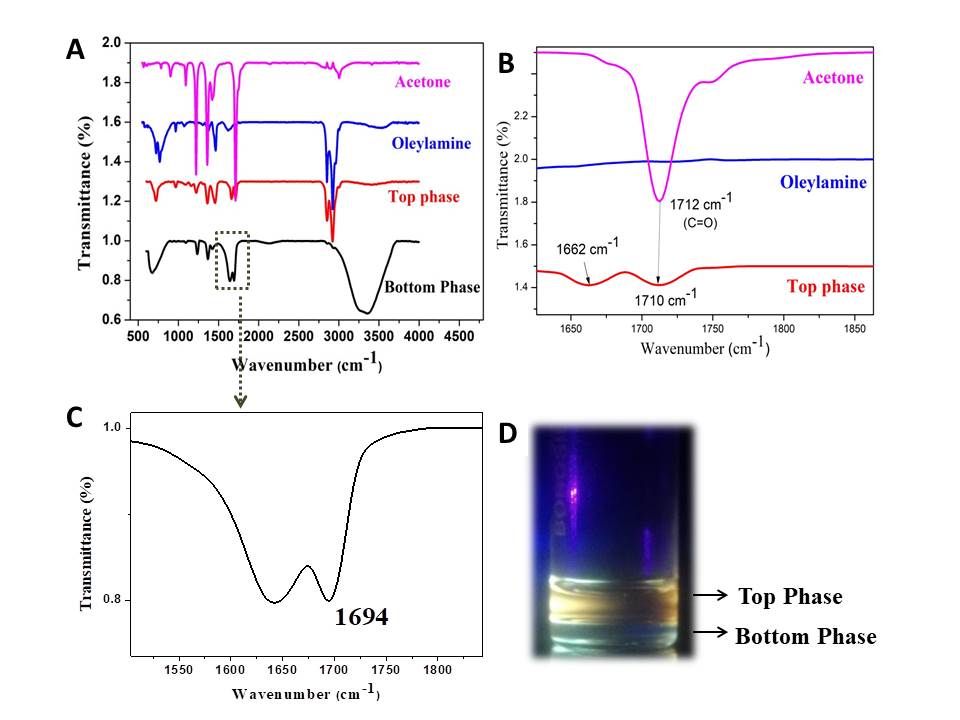


**Fig. S4** (A) FTIR Spectra indicating the condensation reaction between Oleylamine (C18H37N) and Acetone that leads to the formation of Oleylimine type bond between Oleylamine and Acetone, leaving Water. FTIR spectrum corresponding to the bottom phase of the solution has the signature for the presence of water (B) The top phase contains Oleylimine and hydrogen bonded Oleylamine-Acetone. As a signature slightly red shifted C=O mode at 1710 cm^-1^ was observed indicating the formation of hydrogen bond between Oleylamine-Acetone and also a mode at 1662 cm^-1^ was observed as signature for the presence of Oleylimine bonding between Oleylamine and Acetone. (C) The bottom phase contained hydrogen bonded Water and Acetone complex as evidence. (D) Photograph of aged immiscible Oleylamine-Acetone sample prepared with low Acetone content has two different liquid phases under UV illumination shows a yellow emission at top phase and blue emission at bottom phase, having an interface between the two solutions.

**Theoretical emission spectra**

**
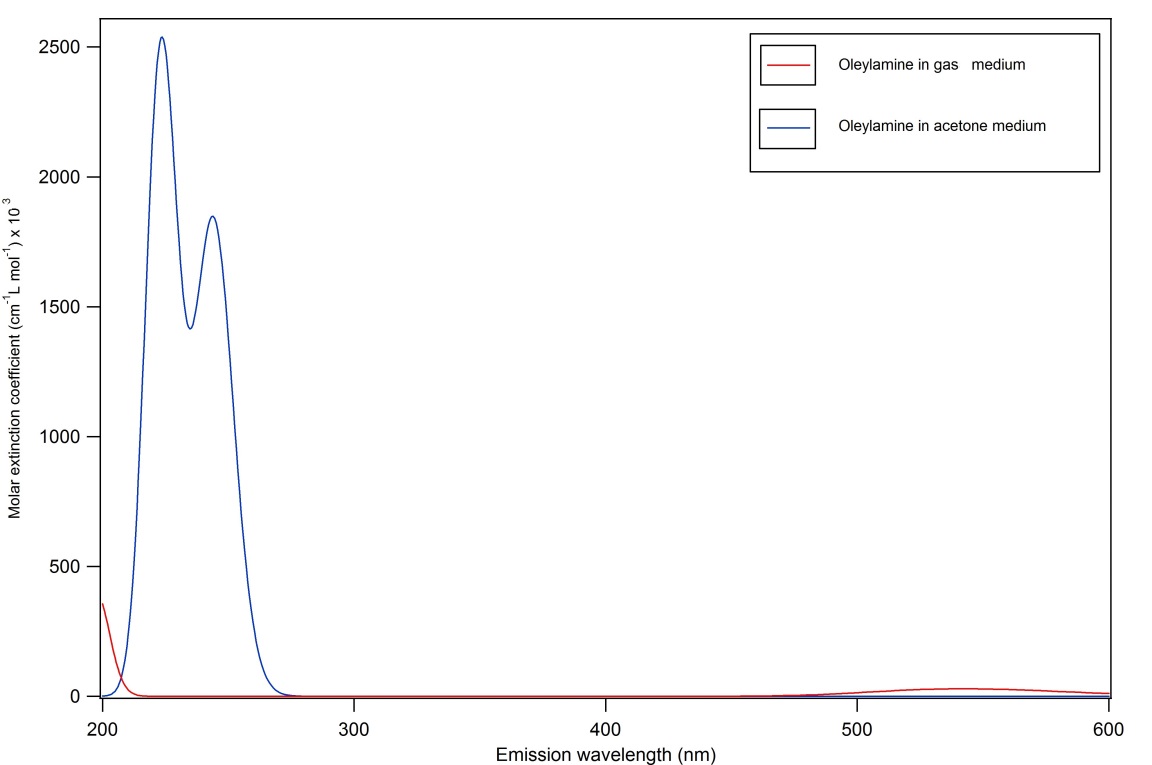
**

**Fig. S5.** Theoretical Emission spectrum of Oleylamine in Gas and Acetone solvent mediums. Oleylamine in Acetone solvent medium exhibited red shifted emission compared to its gas phase spectrumum.

**HOMO, LUMO levels for the studied complexes**

**Table S3. HOMO-LUMO gaps of studied complexes**

| **S. No** | **Species** | **HOMO (eV)** | **LUMO (eV)** | **Energy gap(E_g_)**  **(eV)** |
| --- | --- | --- | --- | --- |
| **1**  **2**  **3**  **4**  **5**  **6**  **7** | OA_OA  OA_Ace1  OA_Ace2  OA_2Ace  Ace_Ace  OA  Ace | -6.25  -6.45  -6.45  -6.20  -6.88  -6.57  -0.26 | 0.47  -0.67  -0.66  -0.73  -0.69  0.47  -0.02 | 6.71  5.78  5.79  5.47  6.21  7.04  0.24 |


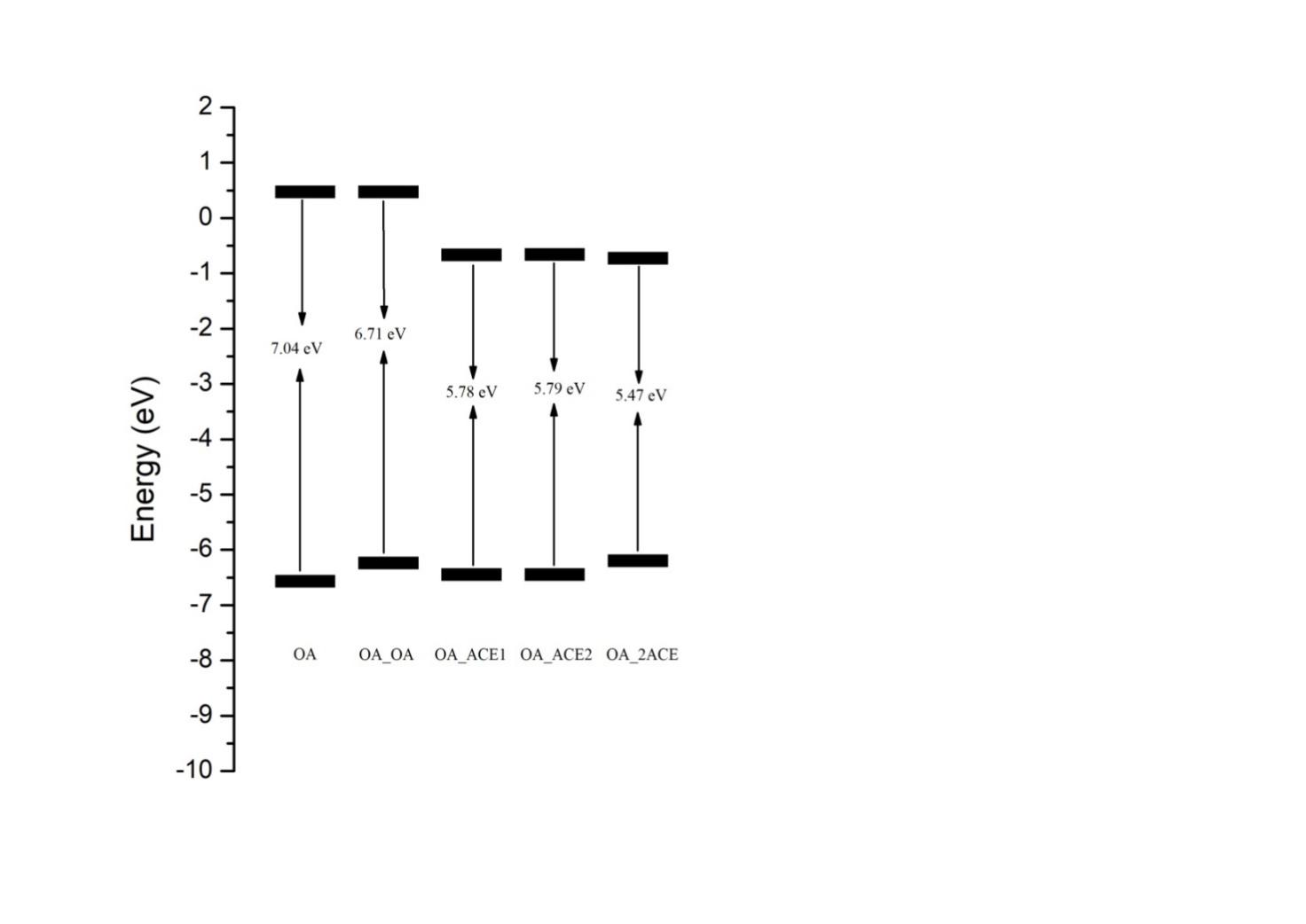


**Fig. S6.** Relative energy level position for the studied complexes (A) Oleylamine, (B) Oleylamine interacting with another Oleylamine, (C) Acetone interacting with hydrogen (H1) of Oleylamine, (D) Acetone interacting with hydrogen (H2) of Oleylamine. (E) Two Acetone molecules interacting with two hydrogen atoms of Oleylamine.

**
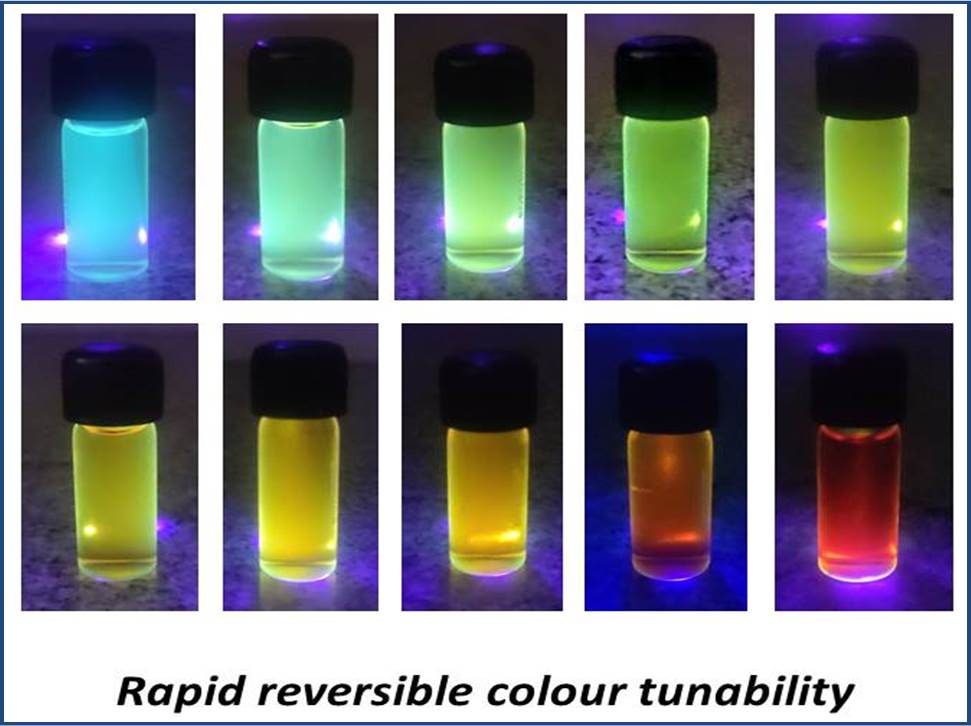
**

**Fig. S7** Photographic image of different dilutions of Oleylamine-Acetone aged samples under UV laser illumination. Rapid red to blue (or) blue to red emission wavelength tunability was possible by varying the dilution level of the solution

**References**

1 Bernacka-Wojcik, I. *et al.* Inkjet printed and “doctor blade” TiO2 photodetectors for DNA biosensors. *Biosensors and Bioelectronics* **25**, 1229-1234, (2010).

2 Kontos, A. I. *et al.* Nanostructured TiO2 films for DSSCS prepared by combining doctor-blade and sol–gel techniques. *Journal of Materials Processing Technology* **196**, 243-248, (2008).
